# Polygala oligosaccharide esters improve memory disorder by restoring gut microbiota homeostasis through the regulation of the “gut-brain” axis

**DOI:** 10.1186/s13020-026-01436-7

**Published:** 2026-06-08

**Authors:** Yan Wang, Chaoyun Ren, Wenkai Dong, Qiule Li, Fanying Deng, Fuxia Zhao, Yangang Cheng, Peng Sun, Huifang Li, Yingli Wang

**Affiliations:** 1 Institute of Pharmaceutical and Food Engineering, Shanxi University of Chinese Medicine, 121 Daxue Road, Yuci District, Jinzhong, 030619 China; 2 Graduate School, Heilongjiang University of Chinese Medicine, 24 He Ping Road, Xiangfang District, Harbin, 150040 China; 3 Department of Neurology, Shanxi Province Integrated Traditional Chinese and Western Medicine Hospital, No. 13 Fudong Street, Taiyuan, 030000 Shanxi China; 4 Experimental Management Center, Shanxi University of Chinese Medicine, 121 Daxue Road, Yuci District, Jinzhong, 030619 China

**Keywords:** Polygala oligosaccharide esters, Memory disorder, Gut-brain axis, Gut microbiota, Lipidomics

## Abstract

**Background:**

Yuanzhi (*Polygala tenuifolia Willd.*) possesses the effects of calming the spirit, enhancing intelligence, regulating the heart-kidney connection, eliminating phlegm, and reducing swelling. It is commonly used in the treatment of insomnia and forgetfulness. Previous studies have indicated that the oligosaccharide esters (OE) derived from Yuanzhi exhibit neuroprotective and memory-enhancing activities. However, its underlying mechanisms, particularly those involving the gut-brain axis, remain unclear.

**Purpose of the research:**

This study aimed to investigate the therapeutic efficacy and underlying mechanisms of Oligosaccharide Esters (OE) from Polygala tenuifolia Willd. against memory dysfunction in mice, with a specific focus on the gut-brain axis.

**Methods:**

A mouse model of memory dysfunction was induced using D-galactose combined with AlCl₃. Behavioral tests, molecular biology techniques (histopathology, enzyme-linked immunosorbent assay (ELISA), immunohistochemistry, and Western blot), and multi-omics approaches (16S rRNA sequencing and lipidomic analysis) were employed to investigate the therapeutic efficacy of OE against memory dysfunction. Meanwhile, with the aid of fecal microbiota transplantation (FMT) assay, we observed the repair of brain and colonic tissues, inflammatory responses and intestinal permeability, further clarified the regulatory effect of OE on gut microbiota, and ultimately revealed the underlying mechanisms of OE mediated by the gut-brain axis.

**Results:**

OE administration significantly enhanced learning and memory in MD mice, repaired neuronal damage in the hippocampal regions (CA1, CA3, DG) of the MD mouse brain, and increased the number of Nissl bodies. OE elevated the serum levels of BDNF and CREB and reduced the TMAO level; simultaneously, it enhanced the activities of SOD and GSH-Px and decreased the MDA content in the brain tissue. OE treatment modulated the relative abundance of the gut microbiota in MD mice, restored the microbial imbalance induced by memory deficits, and particularly affected the abundances of Firmicutes, Bacteroidetes, their ratio (F/B), and genera such as Ligilactobacillus. Lipidomics analysis indicated that OE exerts its therapeutic effects primarily by regulating the glycerophospholipid metabolism pathway, and a total of 17 key differential lipid metabolites were identified. Correlation analysis further revealed that the levels of key differential lipid metabolites, LysoPC(22:2) and PC(38:4), were significantly positively correlated with the levels of neuroprotective factors (CREB, BDNF) and the activities of antioxidant enzymes (SOD, GSH-Px), but were significantly negatively correlated with the harmful metabolite TMAO and the oxidative damage product MDA. In contrast, the lipid metabolite GPEA exhibited a trend opposite to that of LysoPC(22:2) and PC(38:4). Further investigation results demonstrated that OE could repair pathological damage in colon tissue, regulate the levels of the microbial metabolite TMAO and the neurotransmitter 5-HT, reduce the levels of pro-inflammatory factors (LPS, TNF-α, IL-6) in both the brain and colon, and inhibit the abnormal activation of astrocytes and the abnormal hyperphosphorylation of Tau protein. The results of correlation analysis indicated that beneficial bacteria [e.g., Ligilactobacillus) and beneficial lipids (e.g., LysoPC(22:2) and PC(38:4)] were collectively significantly negatively correlated with key pathological indicators (e.g., TMAO and TNF-α) and were positively correlated with the neurotransmitter (e.g., 5-HT). OE also significantly up-regulated the expression of tight junction proteins (Occludin, Claudin-5) in both brain and colon tissues, thereby structurally repairing the damaged gut-brain barrier. FMT experiments showed that FMT improved the learning and memory abilities of mice, repaired neuronal damage in the hippocampus (CA1, CA3, DG), and increased the number of Nissl bodies. In addition, FMT alleviated colonic tissue injury, attenuated inflammatory responses in the brain and colon, and reduced intestinal permeability in MD mice, exerting a therapeutic effect similar to that of OE.

**Conclusion:**

OE exerted anti-amnestic effects via the gut-brain axis, primarily by alleviating neuroinflammation and oxidative stress, restoring gut microbiota homeostasis, and regulating glycerophospholipid metabolism, ultimately improving learning and memory abilities in MD mice.

**Graphical Abstract:**

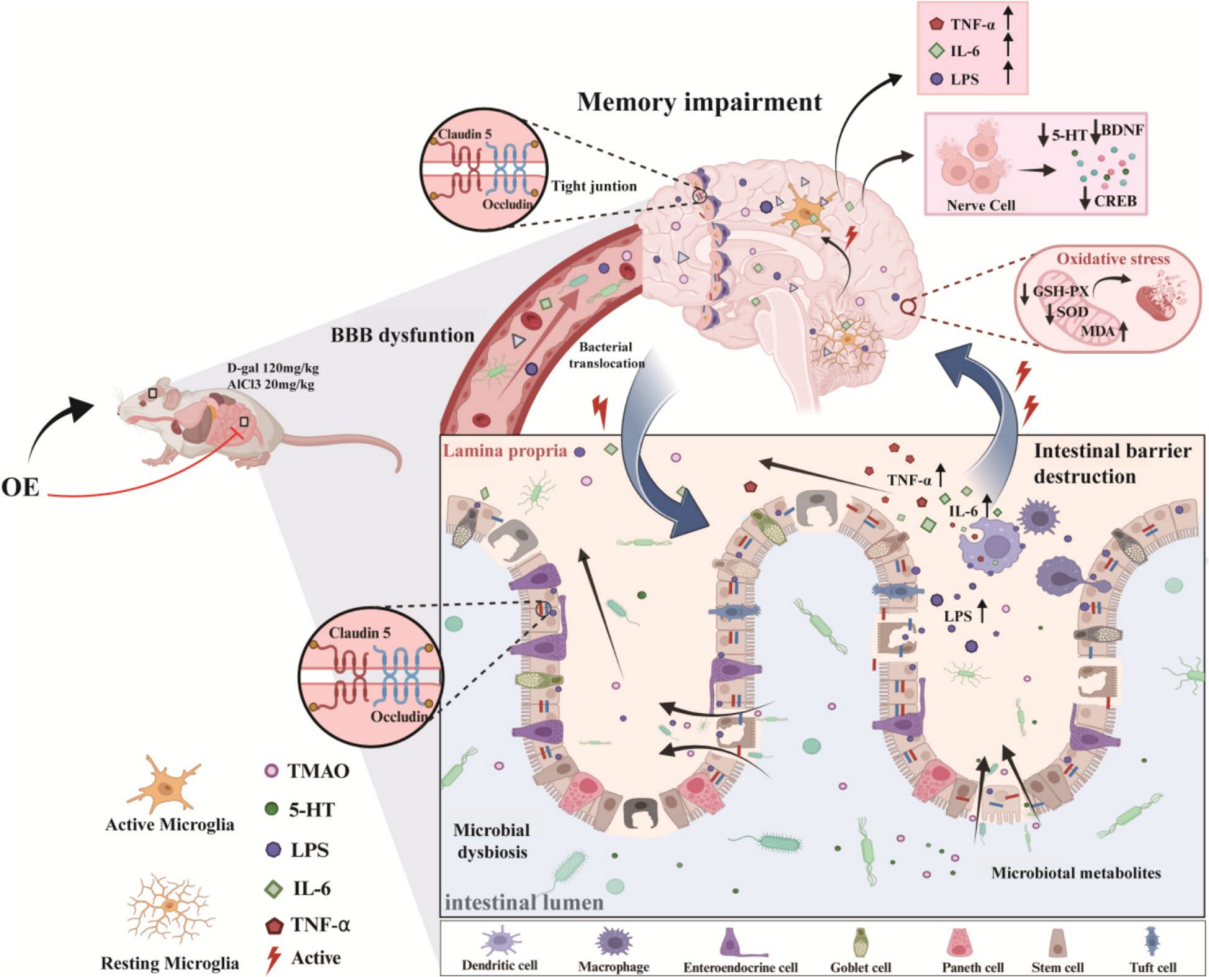

## Introduction

Polygala tenuifolia, a traditional Chinese medicinal plant, is the dried root of *Polygala tenuifolia Willd.* or *Polygala sibirica L.* (Chinese Pharmacopoeia) in the Polygalaceae family. It is recorded in the Chinese Pharmacopoeia that it has the effects of calming the nerves, improving intelligence, connecting the heart and kidneys, expelling phlegm, and reducing swelling. It was recorded in “Shennong’s Classic of Materia Medica”. Its main uses are to treat cough and adverse qi symptoms, replenish the nutrients needed by the body, expel pathogenic factors in the body, ensure the unobstructed flow of the nine orifices, enhance wisdom, make hearing and vision sharper, strengthen memory, improve willpower, and double physical strength. Its main chemical components include saponins, xanthones, and polygala oligosaccharide esters. Modern pharmacological studies have shown that these components have pharmacological effects such as significantly improving learning and memory [1], cognitive function [2], antidepressant effects [3], and anti-inflammatory effects [4]. The effective active component of Polygala tenuifolia, polygala oligosaccharide ester, has been proven to have obvious pharmacological activities such as neuroprotective effects and improvement of memory [5–8]. Moreover, in the classic prescriptions of Polygala tenuifolia, such as Polygala Powder and Dingzhi Xiaowan, it has been found that the components entering the blood all contain polygala oligosaccharide esters, indicating that polygala oligosaccharide esters have obvious pharmacological activities.

MD is a clinical symptom related to multiple diseases, including Alzheimer's disease (AD), cerebral infarction, vascular dementia, post-traumatic stress disorder (PTSD), and neurodegenerative diseases (NDD). Neural disorders are associated with high mortality rates and may result in irreversible neurological deficits. Recent epidemiological studies indicate that the prevalence of cognitive impairment among adults aged 60 years and older in China is approximately 15.5%, with this proportion exhibiting an age-dependent increase. [9]. Memory impairment is closely related to oxidative stress, neuroinflammation, autophagy, mitochondrial dysfunction, apoptosis, and the release and transmission of neurotransmitters and neuroinflammatory factors [10].

Studies have shown that the gut microbiota can conduct dynamic two-way communication through the "gut-brain" axis to regulate the development and function of the immune system, metabolism, and the nervous system [11]. The disorder of the "gut-brain-microbiota" axis may be an important factor in the pathogenesis of neurodegenerative diseases. Changes in the composition of the gut microbiota will lead to an increase in the permeability of the gut barrier and immune activation, which will then trigger a systemic inflammatory response, subsequently damage the blood–brain barrier, cause neuroinflammation, neuron damage, and finally lead to neurodegeneration [12].

In addition, lipids are one of the major components of the cell's bilayer membrane structure and also the first target for diseases to attack nerve cells. Lipid imbalance is closely associated with the occurrence and development of neurodegenerative diseases such as Alzheimer's disease and Parkinson's disease Lipidomics [7, 13, 14], an emerging tool, is used for a comprehensive analysis of all lipid components in biological systems. It can more effectively assist researchers in gaining a deeper understanding of the pathogenesis and therapeutic mechanisms of neurodegenerative diseases, including memory impairment (MD) [10, 15]. Therefore, conducting lipidomic studies on the potential occurrence and development processes of Alzheimer's disease (AD) is beneficial for the early diagnosis of the disease, the elucidation of the pathological mechanisms, and the identification of the drug action targets.

In order to further explore the impact of polygala oligosaccharide ester (OE) on memory impairment, in this study, a memory impairment model induced by D-galactose and aluminum chloride was established in mice. Using 16S rDNA technology, we clarified the role played by microbial metabolites in the process of the extract improving memory impairment. We used lipidomics to explore the impact of memory impairment on the lipid profile. Subsequently, based on the "gut-brain axis" theory, we explored the mechanism of action of the extract in improving memory impairment.

## Materials and methods

### Preparation of OE

Polygala, collected from Xinjiang, Shanxi (20210915), was stored in the Modern Chinese Medicine Engineering Laboratory of Shanxi University of Chinese Medicine. The roots were identified as Polygala tenuifolia Willd. by Lecturer Tan Jinyan from Shanxi University of Chinese Medicine. The roots were pulverized into coarse powder and extracted with a 60% ethanol solution at a tenfold volume, with each extraction lasting 2 h. The extracts were filtered, and the solutions were combined. After solvent recovery and vacuum concentration, the concentrate was centrifuged, and the supernatant was adsorbed on D101 macroporous resin and eluted with a 30% ethanol solution. After the loading process was completed, the adsorption was allowed to stand for 1 h before being washed with water at a flow rate of 3 BV/h to remove impurities. After washing, the resin was eluted with a 30% ethanol solution at a flow rate of 3 BV/h. The eluate was collected, the solvent was recovered, and the solution was concentrated under reduced pressure to dryness to obtain the dry powder of OE, which was stored in a desiccator for future use [16].

The chemical components of OE were analyzed qualitatively using UPLC-Q-TOF-MS/MS with an AB SCIEX system (*USA*). The instrument parameters, chromatographic, and mass spectrometric conditions were as follows. The chromatographic column used was an ACQUITY UPLC BEH C18 (2.1 mm × 100 mm, 1.7 μm) (Waters Associates, Mass, USA), with a flow rate of 0.3 mL/min, an injection volume of 2 μL, and a column temperature of 40 °C. The mobile phase consisted of acetonitrile and 0.1% formic acid aqueous solution, with a gradient elution program as follows: 0–2 min, 5% A; 2–5 min, 5%–10% A; 5–10 min, 10%–30% A; 10–13 min, 30%–60% A; 13–15 min, 60% A; 15–19 min, 60%–100% A; 19–21 min, 100%–5% A; 21–25 min, 5% A. For ESI mass spectrometry in positive and negative ion modes, the conditions were as follows: in positive ion mode, the spray voltage was 3.2 kV, the sheath gas flow rate was 40 arb, the auxiliary gas flow rate was 5 arb, and the auxiliary gas heating temperature was 350 °C; in negative ion mode, the spray voltage was 2.5 kV, the sheath gas flow rate was 38 arb, the auxiliary gas flow rate was 10 arb, and the auxiliary gas heating temperature was 300 °C. The ion transfer tube temperature was set to 320 °C, and the lens voltage (S-Lens RF level) was 50 V. For full scan/dependent data secondary scanning (full MS/dd-MS2), the scan range was m/z 120–1500, with a primary mass resolution of 70,000 FWHM and a secondary resolution of 17,500 FWHM. A collision energy of 30 eV was used. By searching databases such as CNKI, SciFinder, PubMed, and the OE chemical component library, a comprehensive OE chemical component library was established. This library included key information such as compound names, molecular formulas, and relative molecular masses. The precise molecular mass, relative retention time (tR), quasi-molecular ion peaks, and secondary fragment ions provided by Xcalibur 3.0 were compared with the compound information in the reference database to identify the chemical components in OE.

### Animal

SPF-grade male KM mice, 60 in total, 4 weeks old, with a body weight of (20 ± 2) g, were purchased from SPF Biotechnology Co., Ltd. (Beijing, China) (Certificate No.: 110324220103859763, License No.: 20103859763, SCXK (Beijing) 2019–0010). The mice were housed under standard laboratory conditions with a 12-h light/dark cycle and had free access to food and water. All experimental protocols adhered to the Guide for the Care and Use of Laboratory Animals (8th edition). The study was approved by the Animal Ethics Committee of Shanxi University of Chinese Medicine (Approval No.: 2022DW264).

### Animal experimentation

Before the start of the experiment, the mice were allowed to acclimate to their living environment for 1 week and were then divided into 6 groups (*n* = 10) based on body weight. Except for the control group, the mice in the other groups were intraperitoneally injected with D-galactose (120 mg/kg) (Batch No.: bcl-2, C13665374, purity 98%, Shanghai Meilin Biochemical Technology Co., Ltd., China) and administered aluminum chloride (20 mg/kg) (Batch No.: 20220820, purity 97%, Komio Chemical Reagent Factory, Tianjin, China) by gavage for 60 consecutive days. Starting from the 30th day, the OE group mice received different doses of OE by gavage (low dose: 28.80 mg/kg, medium dose: 57.60 mg/kg, high dose: 115.20 mg/kg, once daily) [17], while the piracetam group received a suspension of piracetam tablets (T19C018, 0.4 g, Minsheng Pharmaceutical Co., Ltd., Hangzhou, China) by gavage (0.96 g/kg, once daily). The model group received physiological saline by gavage (same volume as OE, once daily) for 30 consecutive days. The doses of OE and piracetam were calculated based on the conversion ratio of surface area between humans and mice, which is 12:1.

### Behavioral science

Cognitive function assessment was conducted using the Tai Ming Behavioral Analysis System (TM-Vision) (Chengdu, China). The Morris water maze (*MWM*) test was employed to assess learning and memory abilities, with behavioral experiments conducted within 30 min after model construction.

In the MWM experiment, a circular swimming apparatus (120 cm × 60 cm) was used. The water temperature was maintained at 20 ± 2 °C, and a transparent circular platform with a diameter of 15 cm was placed in the second quadrant. Warm water was added to the pool until it was 1 cm above the platform.

For the spatial navigation experiment, the mice in each group were administered the drug 30 min before the experiment and placed at a fixed position in the test pool to acclimate to the environment. The mice were then placed face-down in the pool at the starting position and released into the water in the order of quadrants 1 → 3 → 4, with each quadrant exploration time set to 90 s.

The parameters analyzed included escape latency. After 4 days of continuous training, the platform was removed on the fifth day for the spatial exploration experiment. The mice in each group were placed into the pool from the fourth diagonal quadrant, and their behavior was observed for 90 s. The software automatically recorded the movement trajectories of the mice, the number of entries into the original platform location, and the duration of stay.

A comprehensive analysis and evaluation of the spatial learning and memory abilities of the mice were conducted through the spatial navigation and spatial exploration experiments [18].

### Histopathological observation of brain and colon tissues

After the behavioral tests, three mice from each group were anesthetized with isoflurane (batch number: 1) using a small animal anesthesia machine (batch number: R510-22-10; R500, Reward Life Technology Co., Ltd., Shenzhen, China) and underwent cardiac perfusion. The brain and colon tissues were fixed in a 4% paraformaldehyde solution, embedded in paraffin, and sectioned. Nissl staining (batch number: G1032, Wuhan Saivier Service Biotechnology Co., Ltd., China) was used for brain tissue examination, and hematoxylin and eosin (H&E) staining (batch number: G1005, Wuhan Saivier Service Biotechnology Co., Ltd., China) was used for colon tissue observation.

### Determination of TMAO, CREB, and BDNF expression in serum

Blood samples from each group (*n* = 6) were allowed to stand at room temperature for 30 min, followed by centrifugation at 3500 rpm for 15 min to obtain the supernatant. Trimethylamine N-oxide (TMAO, batch number: F30661-A), cAMP response element-binding protein (CREB, batch number: F2781-A), and brain-derived neurotrophic factor (BDNF, batch number: F2204-A, FANKEWEI Biotech Co. Ltd., Shanghai, China) were quantified using enzyme-linked immunosorbent assay (ELISA). A standard curve was established using a MULTISKAN FC microplate reader (Thermo Fisher Scientific Inc., Shanghai, USA) to calculate the levels of TMAO, CREB, and BDNF in the mouse serum.

### Determination of expression of oxidative stress markers in brain tissue

Mouse brain tissue (*n* = 6) was homogenized at a weight-to-volume ratio of 1:9 in pre-cooled saline to form a 10% brain tissue homogenate. After centrifugation at 4500 rpm for 10 min, the supernatant was collected. Superoxide dismutase (SOD, batch number:  A003-1-2), glutathione peroxidase (GSH-PX, batch number: A005-1-2), malondialdehyde (MDA, batch number:  A003-1-2)were measured using a MULTISKAN FC microplate reader(Jiancheng Bioengineering Institute, Nanjing, China).

### 16S rDNA sequencing

In a sterile environment, fecal samples from mice (*n* = 6) were collected and placed in EP tubes. After liquid nitrogen quenching, the samples were sent to Baiqu Biotechnology Co., Ltd. (Shanghai, China) for 16S rDNA sequencing analysis.

For high-throughput 16S rDNA sequencing analysis of animal gut microbiota, total microbial DNA was extracted from colonic content samples using the CTAB method, eluted with 50 μL buffer solution, and stored at -80 °C. Using primers 341F (5'-cctacgggnggcwggcag-3') and 805R (5'-GACTACHVGGGTATCTAATCC-3'), bacterial 16S rDNA genes in the V3-V4 hypervariable regions were amplified by PCR (A200, Longgene Scientific Instrument Co., Ltd., Hangzhou, China). PCR conditions were as follows: denaturation at 98 °C for 30 s, followed by 32 cycles of denaturation at 98 °C for 10 s, annealing at 54 °C for 30 s, and extension at 72 °C for 45 s, with a final extension at 72 °C for 10 min. Unique barcodes were assigned to the samples for paired-end read length, and barcodes and primer sequences were trimmed. Double-end read data were merged using FLASH software, and filtering was performed using fqtrim (v0.94) to retain high-quality and usable reads. Chimeric sequences were removed using Vsearch software (v2.3.4). DADA2 was then used to process the reads and generate feature tables and feature sequences. Alpha and beta diversity were normalized to the same sequence, and feature abundance was normalized based on the relative abundance of each sample using the SILVA (v138) classifier. QIIME2 (http://qiime2.org/) was used to calculate the Chao1 index, Goods_coverage index, Shannon index, and Simpson index for species diversity and complexity analysis, as well as beta diversity analysis. Finally, each representative sequence was annotated by BLASTing against the SILVA database (http://www.arb-silva.de/).

### Lipidomics research

Analysis was conducted using a UHPLC-MS system (Q Exactive, Thermo Scientific). Preprocessed serum samples (5 µL) were injected into an ACQUITY UPLC BEH C18 column (2.1 × 100 mm, 1.7 µm) and analyzed at 40 °C with a flow rate of 0.3 mL/min. The mobile phase was composed of acetonitrile (A) and 0.1% formic acid in water (B). A gradient elution was applied as follows: 0–2 min, 5% A; 2–5 min, 5–10% A; 5–10 min, 10–30% A; 10–13 min, 30–60% A; 13–15 min, 60% A; 15–19 min, 60–100% A; 19–21 min, 100–5% A; 21–25 min, 5% A. The column was re-equilibrated under 5% A. Electrospray ionization (ESI) was used in both positive and negative ion modes. The spray voltage was set to 3.2 kV. The sheath and auxiliary gas flow rates were 40 and 5 arb, respectively, with the auxiliary gas heated to 350 °C. The ion transfer tube temperature was maintained at 320 °C, and the S-Lens RF level was 50 V. The mass spectrometer was set to a scan range of m/z^−1^ 100–1000 with a collision energy of 30 eV. The LC–MS data were processed using Compound Discoverer 3.3 for peak deconvolution, alignment, calibration, and normalization. The normalized peak area data from all groups were then subjected to multivariate statistical analysis in SIMCA 14.1-P, including principal component analysis (PCA) and orthogonal partial least squares-discriminant analysis (OPLS-DA). The OPLS-DA model was rigorously evaluated using R^2^Y and Q^2^ values, along with permutation testing (R^2^ and Q^2^ intercepts), to ensure robustness and prevent overfitting. Differential metabolites were selected based on a variable importance in projection (VIP) score > 1 from the OPLS-DA model and a *P* < 0.05 from Student's t-test, comparing both the blank versus model groups and the OEM versus model groups. These metabolites were identified by querying databases such as Lipid MAPS and HMDB, and potential biomarkers were proposed. Their expression trends across groups were visualized by cluster heatmaps, while correlations among the putative lipid biomarkers were assessed to elucidate interrelationships. Pathway enrichment analysis of the differential metabolites was performed using the Metabo Analyst 5.0 online platform.

### Determination of TMAO, 5-HT, and inflammatory factor expression in brain and colon tissues

Brain and colon tissues (*n* = 6) were homogenized in phosphate-buffered saline (PBS) at a weight-to-volume ratio of 1:9 to obtain a 10% tissue homogenate. After centrifugation at 4500 rpm for 10 min, the supernatant was collected. Levels of trimethylamine N-oxide (TMAO, batch number: F30661-A), 5-hydroxy-L-tryptophan (5-HT, batch number: F2443-A), lipopolysaccharide (LPS, batch number: F2132-A), tumor necrosis factor-alpha (TNF-α, batch number: F2631-A), and interleukin-6 (IL-6, batch number: F2163-A) were measured using the ELISA method (Vangen Biotech Co., Ltd., Shanghai, China).

### Immunohistochemical detection of the expressions of p-Tau and GFAP in brain tissue

Paraffin-embedded brain tissue sections were prepared. Following deparaffinization and hydration through a graded series of xylene, absolute ethanol, 95% ethanol, and 85% ethanol, antigen retrieval was conducted using sodium citrate buffer. The sections were then blocked with serum and incubated overnight at 4 ℃ with primary antibodies against p-Tau and GFAP (diluted 1:75). After washing with PBS, the sections were incubated with a secondary antibody for 3 h. Color development was performed using DAB, followed by counterstaining with hematoxylin. Finally, the sections were dipped in 1% acid alcohol for 5 s, dehydrated through a graded ethanol series, and mounted with coverslips.

### Determination of tight junction proteins in brain and colon tissues

Frozen brain and colon tissues were weighed and placed in a grinding tube. The tissues were homogenized in RIPA lysis buffer with proteinase and phosphatase inhibitors at a weight-to-volume ratio of 1:9. Grinding beads were added to the mixture, which was then processed in a grinding machine (Wuhan Servicebio Technology Co., Ltd., Wuhan, China). The tissue protein mixture was removed, dissolved on ice for 10 min, and then centrifuged at 4 °C at 12,000 rpm for 15 min. The supernatant was transferred to a new centrifuge tube and stored at − 80 °C. After lysis, homogenization, and centrifugation, the total protein was extracted and quantified using the BCA assay. Proteins were denatured and samples were prepared. SDS-PAGE electrophoresis was performed with an 8% separation gel and a 5% stacking gel. The stacking gel was run at 80 V, and the separation gel at 120 V. The PVDF membrane was wetted for 90 min, blocked with a blocking solution for 15 min, and incubated overnight at 4 °C with primary antibodies (Occludin 1:1000; Claudin 5 1:500; β-actin 1:2000). The membrane was washed three times with TBST for 5 min each, and then incubated with secondary antibodies (goat anti-rabbit 1:6000, 1:4000) at room temperature for 30 min. The membrane was washed again with TBST and visualized with ECL. The bands were analyzed.

### Aluminum trichloride combined with D-galactose induces a mouse model of memory impairment and administration via fecal microbiota transplantation

To further verify the mediating mechanism of the gut-brain axis and clarify the relationship between gut microbiota and cognitive impairment, fecal microbiota transplantation (FMT) was performed in mice in this study. Prior to the experiment, mice were acclimatized for 1 week and randomized into six groups based on body weight: control, model, model + quadruple antibiotic clearance (MA), model + MA + OE (MAE), model + OE (OE), and model + fecal microbiota transplantation (FMT). Except for the control group, all mice received intraperitoneal injection of D-galactose (120 mg/kg) and intragastric administration of aluminum chloride (20 mg/kg) daily for 60 days. From week 3, MA, MAE, and FMT mice were intragastrically administered antibiotics (neomycin 200 mg/(kg·d), metronidazole 200 mg/(kg·d), vancomycin 100 mg/(kg·d), ampicillin 200 mg/(kg·d)) to establish a pseudo-sterile environment. From week 4, MAE and OE mice received intragastric OE (115.20 mg/kg), FMT mice received fecal bacterial suspension, and Model and model + MA mice received normal saline (same dose as OE, once daily) for 30 days. Fecal suspension was prepared by homogenizing fecal contents from OE mice in sterile saline (200 mg feces/2 mL saline), filtering through sterile gauze, centrifuging at 1200 r/min for 5 min, and adjusting the supernatant to the original volume.

### Behavioral science

The behavioral tests were performed according to the procedures described in Sect. “Behavioral science”.

### Histopathological observation of brain and colon tissues

Brain tissues and colon tissues were fixed with 4% paraformaldehyde, then the following steps were performed: dehydration, embedding in paraffin, slicing, haematoxylin and eosin (H&E) staining and Nissl staining for pathological evaluation.

### Expression of LPS, TNF-α and IL-6 in brain and colon tissues

The enzyme-linked immunosorbent assay (ELISA) kits used to detect the levels of mouse LPS, TNF-α, IL-6 in brain and colon tissues were purchased from Winshare Bio-Tech (Shanghai) Co., Ltd. The experimental procedures were carried out according to the manufacturer’s instructions, which were consistent with those described in Sect. “Determination of TMAO, 5-HT, and Inflammatory Factor Expression in Brain and Colon Tissues” of this study. The concentrations of target proteins were determined by standard protein curves.

### Permeability of FITC-dextran

All mice were fasted for 12 h before receiving an oral dose of 50 mg/kg of FITC-dextran (FD-4,4kDa, A1149843, Ambeed). After 4 h, the distribution of FITC-dextran in mice was observed using a small animal imaging system (IVlS Lumina Series lll, America).

Peripheral blood was collected from mice and allowed to stand for 30 min in the dark. The samples were centrifuged at 860 g at 4 ℃ for 10 min to collect serum. The fluorescence intensity of the serum was detected, and a standard curve was used to calculate the concentration of FITC-dextran in the serum.

### Statistical methods

The experimental data were processed using SPSS 27.0 (SPSS, Inc., Chicago, IL, USA), GraphPad Prism 8.0 (San Diego, California, USA) and Origin 2021 (OriginLab, Inc., Northampton, MA, USA). Results were expressed as mean ± SD. Data with a normal distribution were analyzed using one-way analysis of variance (ANOVA). Data with a non-normal distribution were analyzed using non-parametric tests (Kruskal–Wallis test). Spearman's correlation analysis was employed to examine the correlation between differential microbiota and biochemical indicators. A *p*-value < 0.05 was considered statistically significant.

## Results

### Qualitative analysis of chemical components* in OE*

**Fig. 1:**
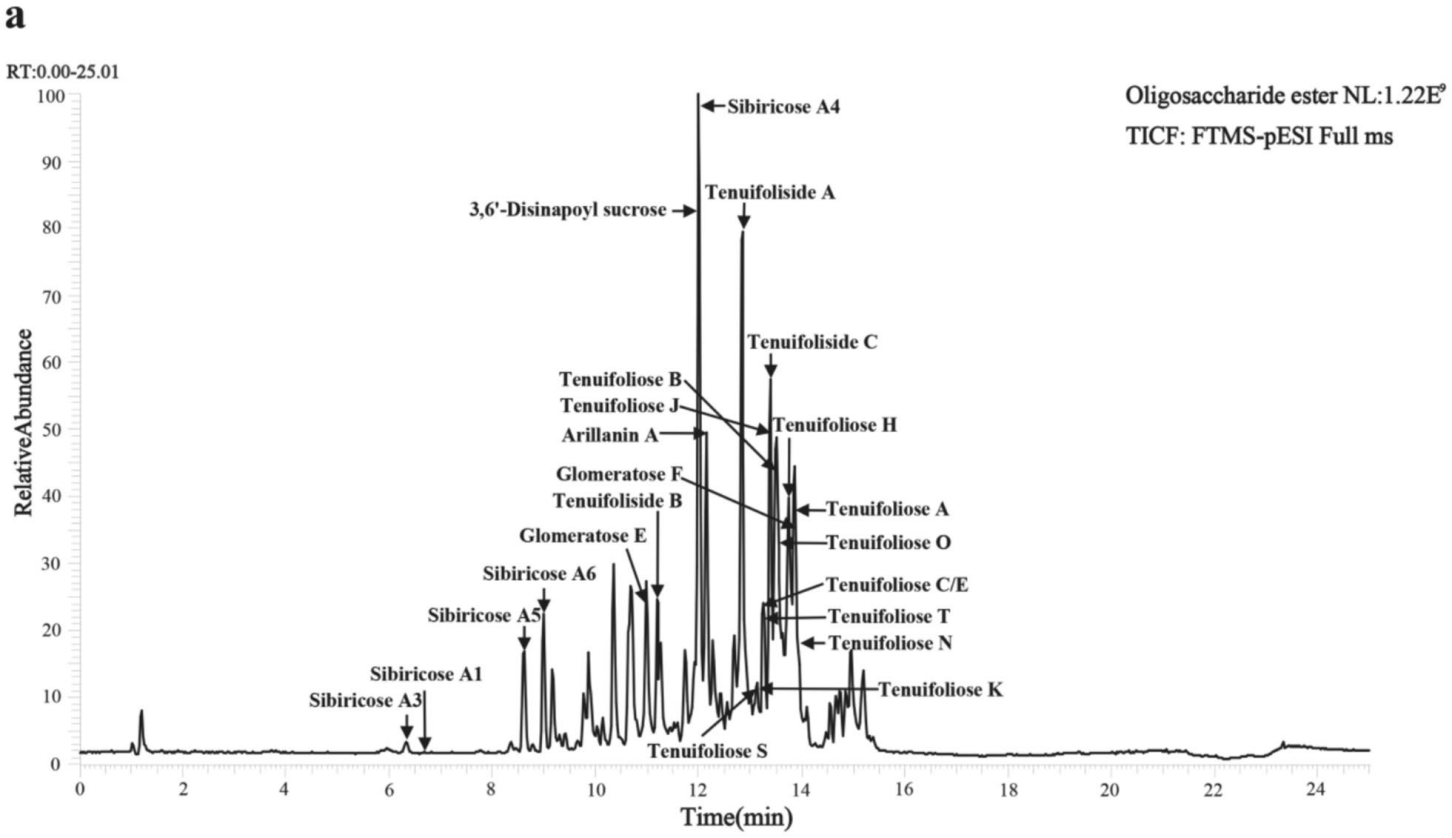
Negative ion mode of polygala oligosaccharide ester

The total ion chromatogram (Fig. 1a) was used to extract mass spectrometry information from the OE extract and compare it with literature references. A total of 22 chemical components were identified, including Sibiricose A3, Sibiricose A5, Tenuifoliside A, Tenuifoliside C, Sibiricose A6, and 3,6'-diallyl sucrose (Table 1). Chromatographic peaks were marked for all components, including Sibiricose A5, 3,6'-diallyl sucrose, and Tenuifoliside A.

**Table 1:** Qualitative results of OE components

No.	Compound	tR(min)	Formula	Calculated mass(*m/z*)	Measured mass(*m/z*)	Mass error/ppm	MS/MS(-)
1	Sibiricose A3	6.33	C_19_H_26_O_13_	461.1301	461.1295	− 1.3043	209.0446, 299.0756, 281.0664, 239.0552 [26]
2	Sibiricose A1	6.62	C_23_H_32_O_15_	547.1668	547.1673	− 0.9140	119.0333, 175.0024, 190.0259, 205.0496, 341.0012 [26]
3	Sibiricose A5*	8.62	C_23_H_32_O_15_	517.155	517.1552	0.38684	329.0662, 339.2326, 340.2361
4	Sibiricose A6*	8.99	C_22_H_30_O_14_	547.1659	547.1660	0.18281	175.0021, 190.0258, 205.0495, 223.0602, 367.1028
5	Glomeratose E	10.92	C_34_H_40_O_19_	751.208	751.2083	0.3995	259.0246, 427.1020 [26]
6	Tenuifoliside B	11.19	C_30_H_36_O_17_	667.188	667.1873	− 1.0495	137.0228, 205.0495, 461.1305, 614.8856 [27]
7	3,6'-Disinapoyl sucrose*	11.98	C_34_H_42_O_19_	753.2234	753.2236	− 0.2656	164.0465, 190.0259, 205.0495, 223.0602, 365.0719, 325.0947
8	Sibiricose A4	12.00	C_34_H_42_O_19_	753.2246	753.2236	− 1.3280	205.0492 [26]
9	Arillanin A	12.14	C_33_H_40_O_18_	723.2136	723.2133	− 0.4149	190.0257, 205.0494, 223.0602 [28]
10	Tenuifoliside A*	12.85	C_31_H_38_O_17_	681.2031	681.2025	− 0.8810	127.0228, 223.0604, 239.0551, 443.1120,
11	Tenuifoliside C*	13.39	C_35_H_44_O_19_	767.2399	767.2394	− 0.6519	205.0495, 237.0764, 325.0944, 367.1041, 529.1581
12	Tenuifoliose S	13.10	C_55_H_68_O_31_	1223.3671	1223.3646	− 2.0442	145.0278, 307.0817, 955.2739 [26]
13	Tenuifoliose K	13.12	C_57_H_70_O_32_	1265.3777	1265.3751	− 2.0553	425.6346, 997.2932, 1119.3353 [27]
14	Tenuifoliose C/E	13.26	C_58_H_72_O_33_	1295.3883	1295.3849	− 2.6255	851.2659, 1119.3464, 997.3049 [27]
15	Tenuifoliose T	13.28	C_56_H_70_O_32_	1253.3772	1253.3748	− 1.91540	307.0821, 631.1880, 647.1964, 955.2874 [28]
16	Tenuifoliose J	13.37	C_59_H_72_O_33_	1307.3878	1307.3849	− 2.21882	307.0814, 339.0854, 631.1878, 689.2134, 997.3006, 1119.3443 [28]
17	Tenuifoliose B	13.48	C_60_H_74_O_34_	1337.3983	1337.3949	− 2.5430	307.0819, 339.0857, 631.1868, 997.3057, 1119.3494 [28]
18	Tenuifoliose O	13.60	C_61_H_76_O_35_	1367.4094	1367.4056	− 2.77981	1027.3105 [26]
19	Tenuifoliose H	13.80	C_61_H_74_O_34_	1349.3983	1349.3953	− 2.22387	674.2025, 731.2242, 1039.3158, 1161.3380 [29]
20	Glomeratose F	13.85	C_54_H_64_O_29_	1175.3461	1175.3441	− 1.7021	307.0824, 673.1930 [26]
21	Tenuifoliose A	13.87	C_62_H_76_O_35_	1379.4094	1379.4059	− 2.5381	1039.3136, 1161.3585 [27]
22	Tenuifoliose N	13.94	C_63_H_78_O_36_	1409.4200	1409.4176	− 0.9141	1069.3197, 1191.3413 [27]

*It indicates that the identification of the compound has been verified against a standard reference

### OE enhances learning and memory ability

**Fig. 2:**
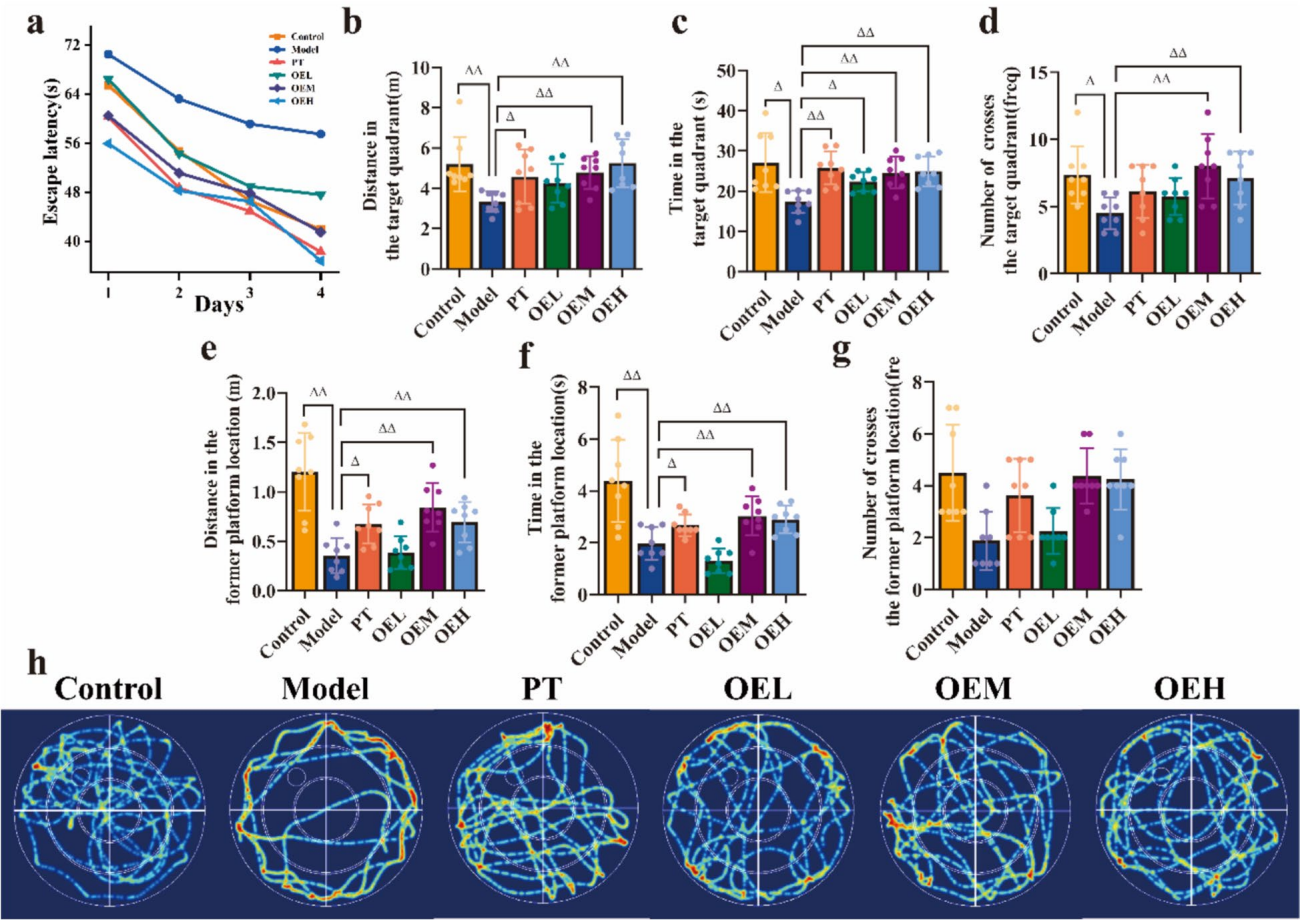
OE can enhance the learning and memory abilities of mice with Memory disorder, repair brain tissue damage, and enhance neuroprotective effects. **a** Morris water maze spatial exploration test. **b** Distance moved in the target quadrant. **c** Time spent in the target quadrant. **d** Number of entries into the target quadrant. **e** Number of entries into the original platform. **f** Time spent in the original platform. **g** Distance moved in the original platform; *n* = 8, *x̄* ± s, compared with the model group, ^∆^*P* < 0.05, ^∆∆^*P* < 0.01. **h** Track map of the spatial exploration test on the 5th day in the Morris water maze.

In the MWM experiment, after 4 days of spatial navigation training, the escape latency of mice in each group decreased progressively (Fig. 2a). The control group showed the greatest reduction, followed by the PT and OEM groups, indicating that the MD mouse model induced by D-galactose combined with aluminum chloride exhibited spatial MD. On the 5th day of the spatial exploration test (Fig. 2b–g), the model group exhibited a significant reduction in movement distance (*P* < 0.01), stay time (*P* < 0.05), and crossings (*P* < 0.05) in the target quadrant, as well as stay time (*P* < 0.01), crossings (*P* < 0.01), and movement distance (*P* < 0.01) at the original platform location compared to the control group. The OEM and OEH groups showed significant increases in movement distance, crossings, and stay time in the target quadrant and at the original platform location (*P* < 0.01). The OEL group also significantly increased the number of mice entering the target quadrant (*P* < 0.05). The PT group demonstrated significant improvements in stay time and movement distance in the target quadrant, as well as crossings, movement distance, and stay time at the original platform location (*P* < 0.05). These findings suggest that OE treatment markedly improved learning and spatial memory abilities in MD mice.

### OE can repair brain tissue injury

**Fig. 3:**
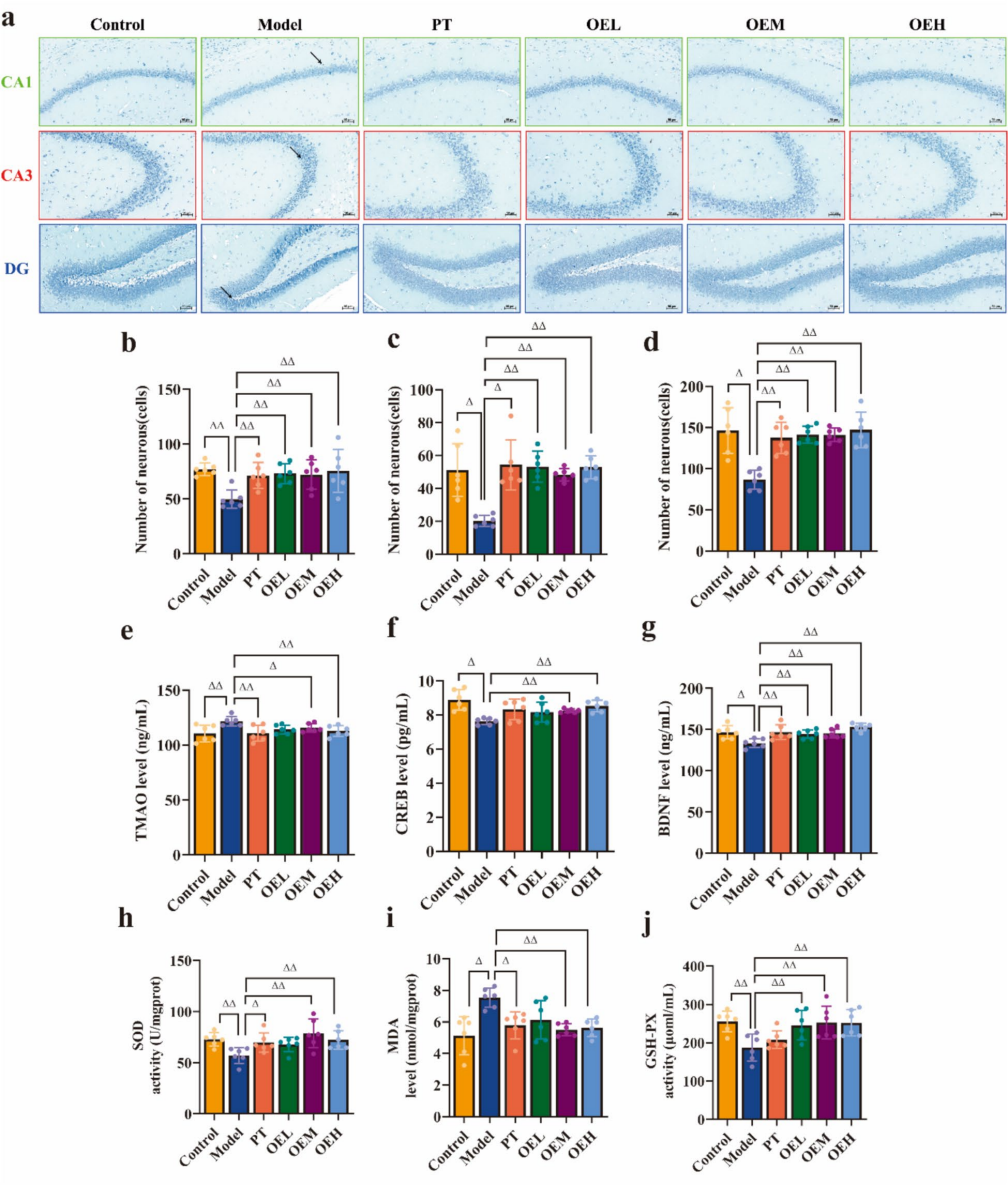
OE can repair brain tissue damage, enhance neuroprotection and improve antioxidant capacity. **a** Nissl staining (× 200); black arrow indicates nuclear pyknosis. **b** Number of neurons in the CA1 region. **c** Number of neurons in the CA3 region. **d** Number of neurons in the DG region; *n* = 3, *x̄* ± s, compared with the model group, ^∆^*P* < 0.05, ^∆∆^*P* < 0.01. **e**–**g** Contents of TMAO, CREB, and BDNF in mouse serum; *n* = 6, *x̄* ± s, compared with the model group: ^∆^*P* < 0.05, ^∆∆^*P* < 0.01. **h**–**j** Activities of SOD, MDA, and GSH-PX in mouse brain tissue; *n* = 6, mean ± standard deviation, compared with the model group: ^∆^*P* < 0.05, ^∆∆^*P* < 0.01.

Nissl staining observations (Fig. 3a) revealed that, compared with the control group, the number of cells and Nissl bodies in the CA1, CA3, and DG regions of the brain were significantly decreased in the model group (*P* < 0.05, *P* < 0.01). Conversely, following treatment with OEL, OEM, and OEH, the neurons were neatly arranged, the number of Nissl bodies increased, and the number of nerve cells also increased (*P* < 0.01) in these regions. The nuclei were stained clearly and evenly, indicating significant neuroprotection by Polygala oligosaccharide esters, which alleviated neurocellular damage in MD mice (Fig. 3b–d).

### OE can enhance neuroprotective effects

In the model group, serum levels of CREB and BDNF were significantly reduced (*P* < 0.01), while TMAO levels were significantly increased (*P* < 0.01) compared to the control group. The PT group significantly increased serum levels of CREB (*P* < 0.05) and BDNF (*P* < 0.01), and reduced the TMAO level (*P* < 0.01). The OEL group significantly increased BDNF levels (*P* < 0.05), while the OEM and OEH groups significantly enhanced CREB and BDNF levels (*P* < 0.05) and simultaneously reduced TMAO levels (*P* < 0.05) (Fig. 3e–g).

### OE can enhance antioxidant stress resistance ability

Compared with the control group, the model group showed a significant increase in MDA content in brain tissue (*P* < 0.05) and a significant decrease in SOD and GSH-PX activities (*P* < 0.05 and *P* < 0.01). The PT group also significantly reduced MDA content (*P* < 0.05). The OEL group significantly increased GSH-PX activity (*P* < 0.05), the OEM group significantly increased both SOD (*P* < 0.05) and GSH-PX activities (*P* < 0.01) while reducing MDA levels (*P* < 0.01), and the OEH group significantly increased GSH-PX activity (*P* < 0.01) and reduced MDA levels (*P* < 0.05). These results suggest that OE treatment effectively mitigates neuro- and oxidative damage in the brains of MD mice (Fig. 3h–j).

### The impact of OE on the gut microbiota of MD mice

Previous studies have established a correlation between gut dysbiosis and the occurrence and progression of MD. Pharmacological studies have demonstrated that OE alleviates behavioral deficits, improves biochemical markers, and mitigates pathological changes in brain tissue in MD mice. To further explore how OE regulates the gut microbiota to exert these beneficial effects, this study utilized 16S rDNA gene sequencing and bioinformatics analysis.

#### Quantitative analysis of OTUs between groups

**Fig. 4:**
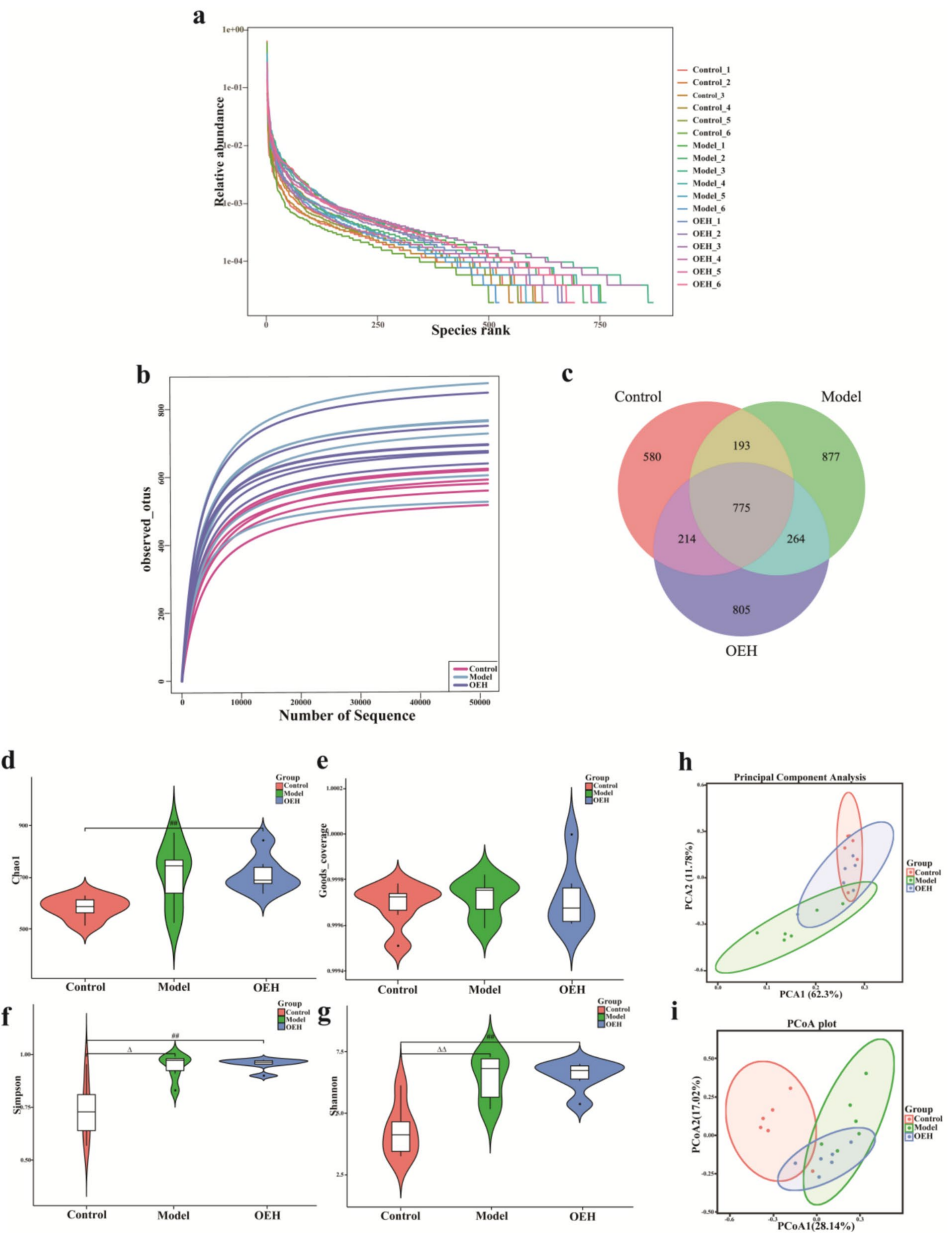
The impact of OE on the gut microbiota of MD mice. **a** Richness rank curve. **b** Rarefaction curve. **c** OTUs Veen analysis. **d**–**g** Alpha diversity analysis. **d** Chao 1 index. **e** Good-coverage index. **f** Simpson index. **g** Shannon index; *n* = 6, *x̄* ± s, compared with the model group: ^∆^*P* < 0.05, ^∆∆^*P* < 0.01; compared with the control group: ^*#*^*P* < 0.05, ^*##*^*P* < 0.01. **h**, **i** Beta diversity analysis. **h** PCA plot. **i** PCoA plot

The sequencing data obtained in this study were of adequate quantity and depth, reliably reflecting the microbial diversity in the fecal samples from each group of mice (Fig. 4a, b). Operational taxonomic units (OTUs) were clustered based on type and quantity in each sample group, and their shared and unique OTUs were analyzed. Venn analysis of OTUs across the three groups revealed a total of 3708 OTUs, including 775 shared OTUs (Fig. 4c). The number of OTUs in the control, model, and OEM groups were 1762, 2109, and 2058, respectively. Each group contained 580, 877, and 805 unique OTUs, respectively. These results indicate a trend towards restoration of OTU numbers in the OE-treated groups, approaching those of the control group, suggesting significant differences in microbial OTUs between the experimental groups.

#### Alpha diversity analysis

Alpha diversity provides insights into the richness, evenness, and sequencing depth of the microbial species detected. The indices used in this analysis include Chao1, which estimates the number of species in the community, and Goods-coverage, which represents the coverage of the microbial community. A higher Goods-coverage value indicates a more accurate reflection of the microbial community. Shannon and Simpson indices measure sample diversity, with higher Shannon values indicating greater diversity and higher Simpson values indicating lower diversity. The results showed that compared with the control group, the Chao1 index in the model group and OEH group increased significantly, with the OEH group showing the most significant increase (*P* < 0.01). There was no significant difference in the Goods-coverage index between the groups. The Shannon index and Simpson index also increased significantly in the model group and OEH group (*P* < 0.05 and *P* < 0.01, respectively), with the OEH group showing a trend towards recovery to levels observed in the control group. These data indicate that, following the induction of MD, the diversity and abundance of gut microbiota increased, with a decreasing trend observed after OEH treatment (Fig. 4d–g).

#### Beta diversity analysis

In addition to alpha diversity, beta diversity assesses the overall diversity or biological heterogeneity of a community in a specific environment, highlighting species differences between groups. Principal Coordinate Analysis (PCA) demonstrated that samples within each group were relatively clustered, while samples between groups were distinctly separated, indicating significant differences among the groups. Notably, compared with the model group, the intestinal microbiota structure of the OEH group showed a trend towards convergence with that of the control group. Principal Coordinates Analysis (PCoA) further indicated that the control group and the model group could be independently classified. After OEH administration, the microbiota structure in MD mouse samples approached that of the control group, consistent with the PCA results (Fig. 4h, i).

#### Analysis of gut microbiota composition

**Fig. 5:**
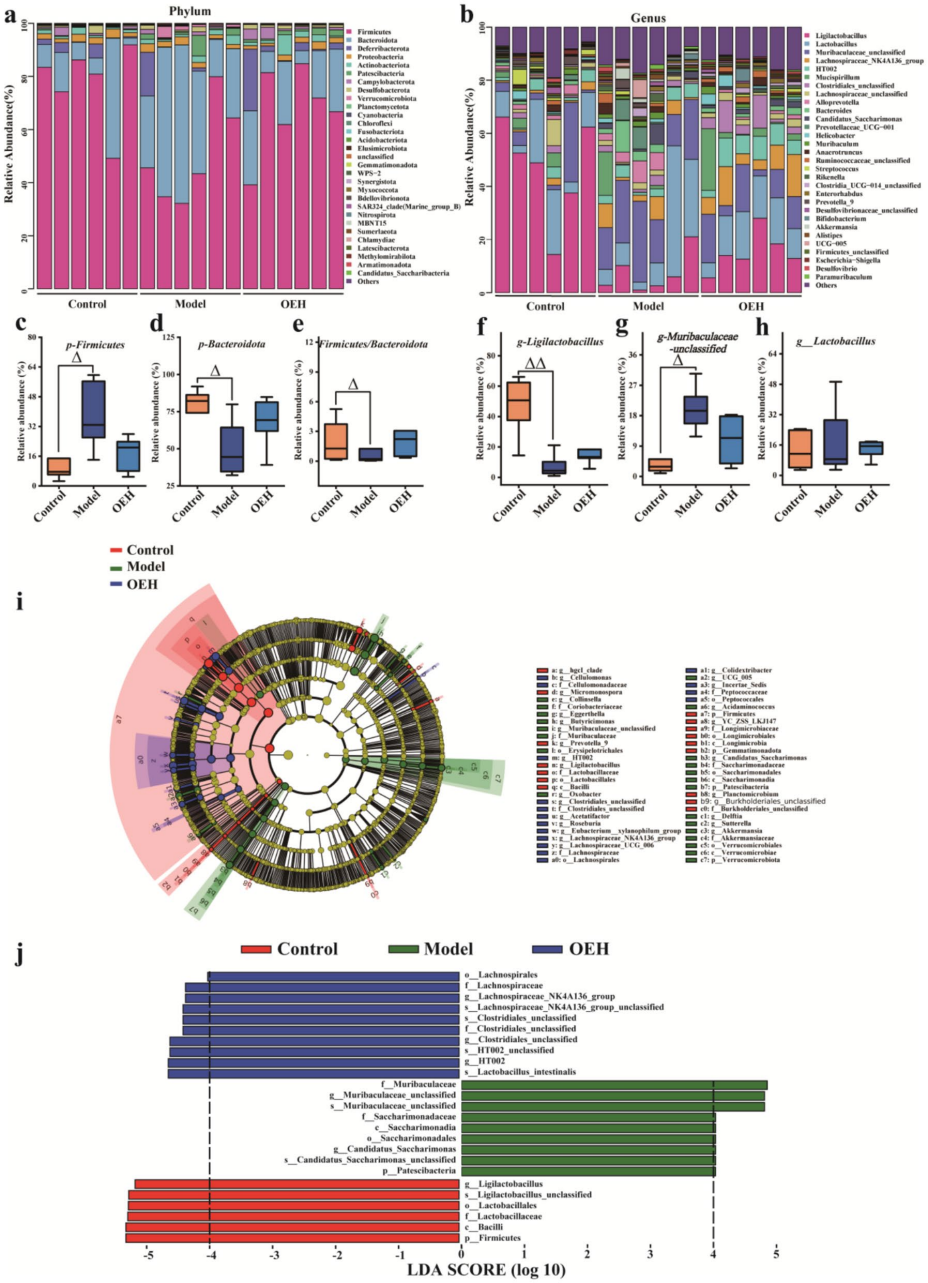
Gut microbiota composition analysis and linear discriminant analysis. **a** Relative abundance of gut microbiota at the phylum level. **b** Relative abundance of gut microbiota at the genus level. **c** Relative abundance of Firmicutes. **d** Relative abundance of Bacterioidota. **e** Firmicutes/Bacterioidota ratio; *n* = 6, *x̄* ± s, compared with the model group: ^∆^*P* < 0.05, ^∆∆^*P* < 0.01. **f** Relative abundance of Ligilactobacillus. **g** Relative abundance of Muribaculaceae_unclassified. **h** Relative abundance of Lactobacillus; *n* = 6, *x̄* ± s, compared with the model group: ^∆^*P* < 0.05, ^∆∆^*P* < 0.01. **i**, **j** Differential species analysis. **i** LEfSe evolutionary branch diagram. **j** LEfSe histogram

Species composition analysis detailed the composition and relative abundance of fecal microbiota at both the phylum and genus levels for each group (Fig. 5a, b). The dominant phyla observed were Firmicutes and Bacteroidetes. Compared to the control group, the model group exhibited a significant reduction in the abundance of Firmicutes (*P* < 0.05) and a significant increase in Bacteroidetes (*P* < 0.05), resulting in a significantly decreased Firmicutes/Bacteroidetes ratio (*P* < 0.05) (Fig. 5c–e). In contrast, the OEH group did not show significant differences in the abundance of Firmicutes, Bacteroidetes, or the Firmicutes/Bacteroidetes ratio compared to the model group. However, the relative abundance levels in the OEH group tended to converge with those of the control group, indicating that OEH treatment could effectively reset the relative abundance of the microbiota. At the genus level, Ligilactobacillus, Lactobacillus, and Muribaculaceae_unclassified were the dominant genera, each with an average relative abundance greater than 5%. Compared to the control group, the model group exhibited a significant reduction in Ligilactobacillus (*P* < 0.01) and a significant increase in Muribaculaceae_unclassified (*P* < 0.05), while the abundance of Lactobacillus did not differ significantly among the groups (Fig. 5f–h). Following OEH treatment, the abundances of Ligilactobacillus, Muribaculaceae_unclassified, and Lactobacillus all showed a trend towards convergence with those observed in the control group, consistent with the findings at the phylum level. These results suggest that OE can restore intestinal microbiota dysbiosis in MD mice by regulating the relative abundance of the intestinal microbiota.

#### Linear discriminant analysis of microbial species

Linear Discriminant Analysis Effect Size (LEfSe) was used to evaluate the impact of sample abundance on microbial variation, with a threshold set at *P* < 0.05 and Lg (LEfSe score) > 4 for identifying biomarkers. A total of 25 marker microbial groups were identified from the phylum to genus levels (Fig. 5i, j). The model group was characterized by specific microbial groups, including the phylum Patescibacteria, the unclassified genus Muribaculaceae, and the genus Candidatus_saccharimonas. Conversely, the control group was marked by the presence of the phylum Firmicutes and the genus Ligilactobacillus. In the OEH group, significant markers included Lachnospiraceae_NK4A136_group, Clostridiales_unclassified, and HT002, with the first two belonging to the Firmicutes phylum. These findings suggest that Firmicutes may serve as a potential biological marker for OE's regulation of the intestinal microbiota.

#### Functional analysis of gut microbial community

**Fig. 6:**
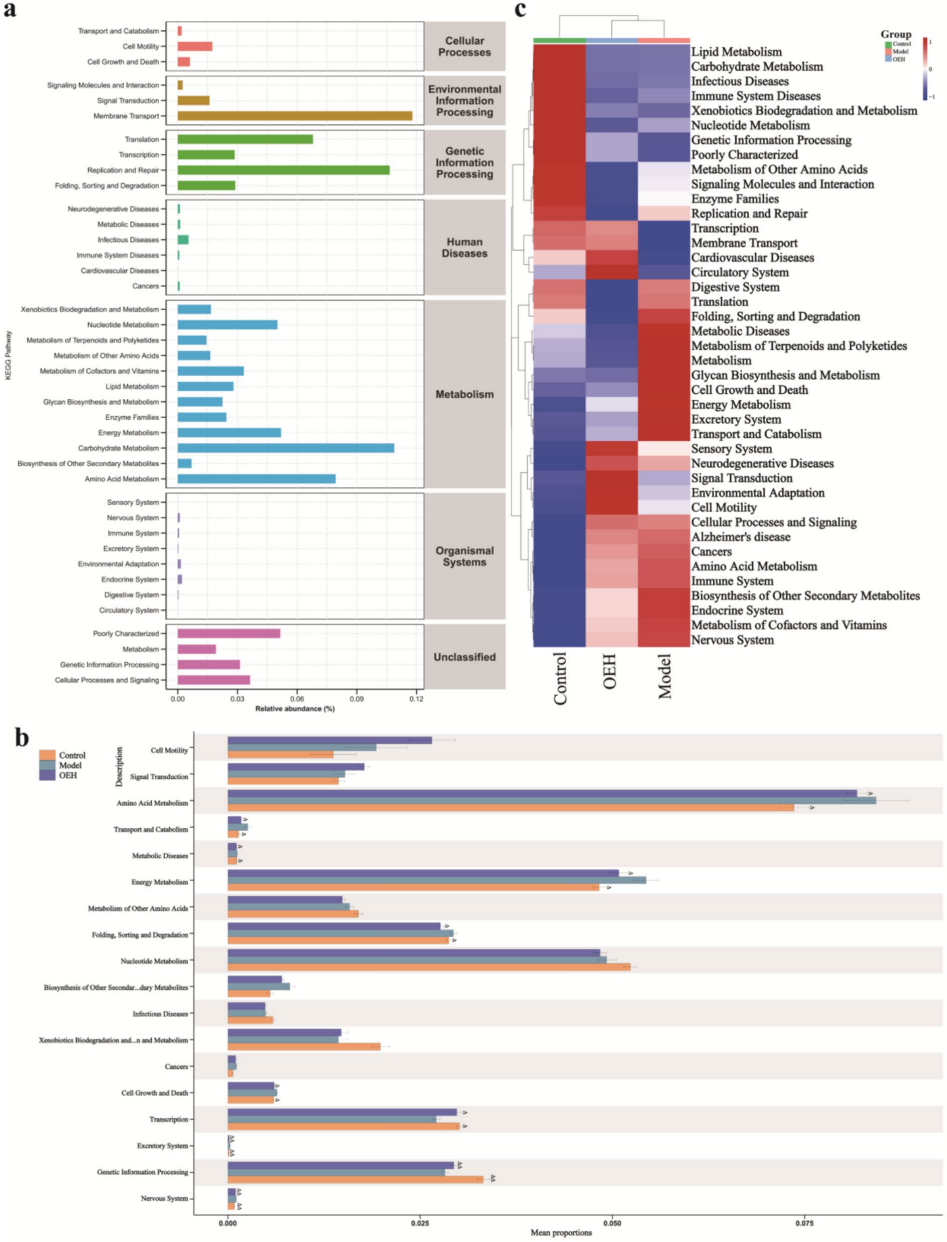
Functional analysis of gut microbial community. **a** Level 1 KEGG functional annotation analysis of differential gut microbiota. **b** Level 2 KEGG functional annotation analysis of differential microbiota. **c** Level 3 KEGG functional annotation analysis of differential microbiota; *n* = 6, *x̄* ± s, compared with the model group: ^∆^*P* < 0.05, ^∆∆^*P* < 0.01

The functional potentials of different OTUs were analyzed using KEGG functional annotations based on 16S rDNA sequencing data (Fig. 6a–c). At the first level of KEGG metabolic pathways, genes related to metabolism, genetic information processing, and environmental information processing exhibited the highest relative abundance. At the second level, genes associated with carbohydrate metabolism, amino acid metabolism, nucleotide metabolism, energy metabolism, and lipid metabolism were most abundant. At the third hierarchical level, the activity of the metabolic pathway related to Alzheimer's disease in MD mice increased, and it was significantly improved after the intervention of OE. In addition, the research results also indicated that the activity of the lipid metabolic pathway was relatively strong in the mice of the blank group, while it would be decreased after induction by D-galactose and aluminum chloride. Additionally, within genetic information processing, functions related to replication, repair, and translation showed high gene abundance, as did membrane transport functions in environmental information processing. This indicates a high abundance of genes involved in these key metabolic and functional processes.

### OE enhances the neuroprotective and anti-oxidative stress abilities of mice with memory impairment by regulating the glycerophospholipid metabolism pathway.

**Fig. 7:**
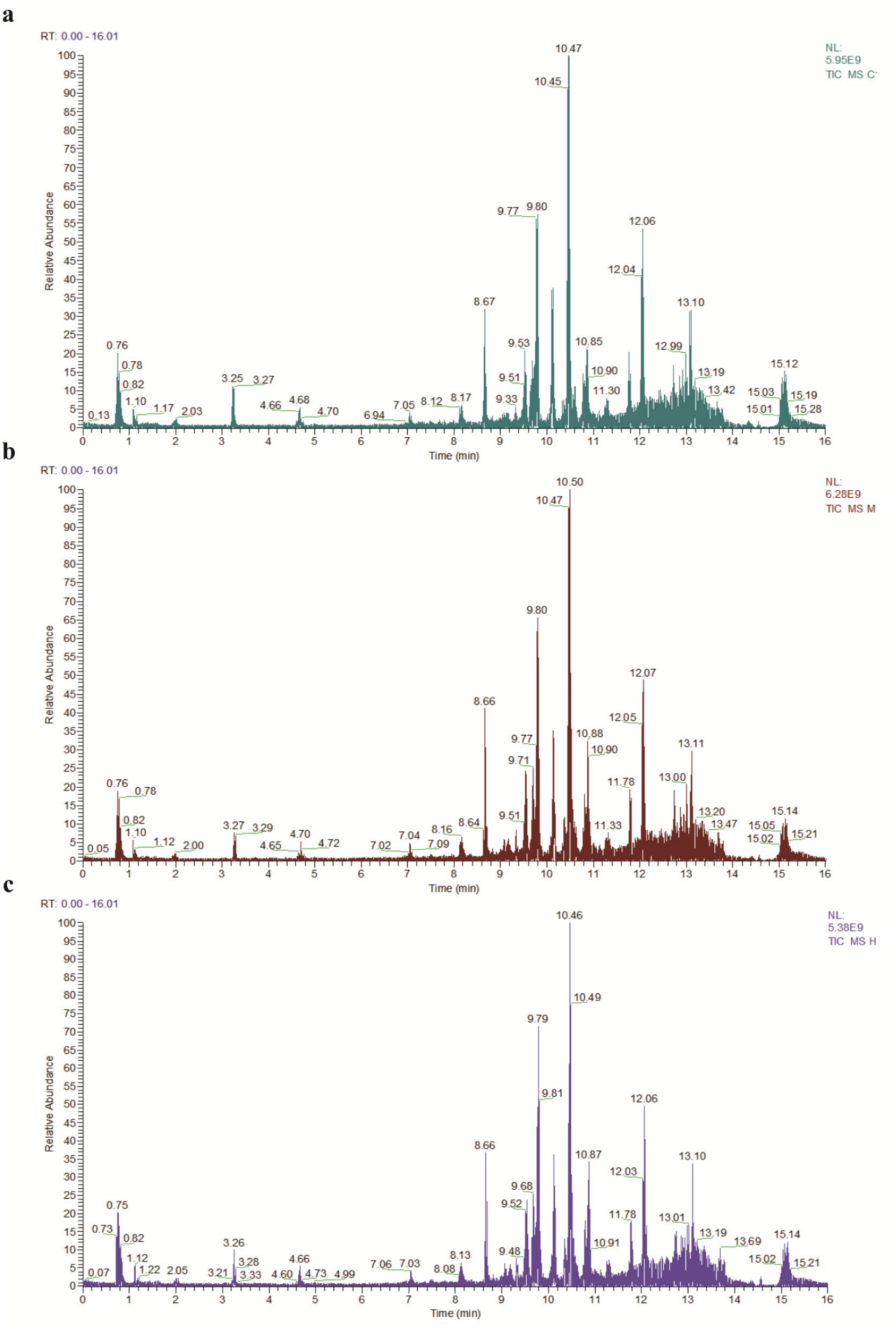
Total ion current chromatogram. **a** Blank group: total ion chromatogram. **b** Model group: total ion chromatogram. **c** OEH group: total ion chromatogram

**Fig. 8:**
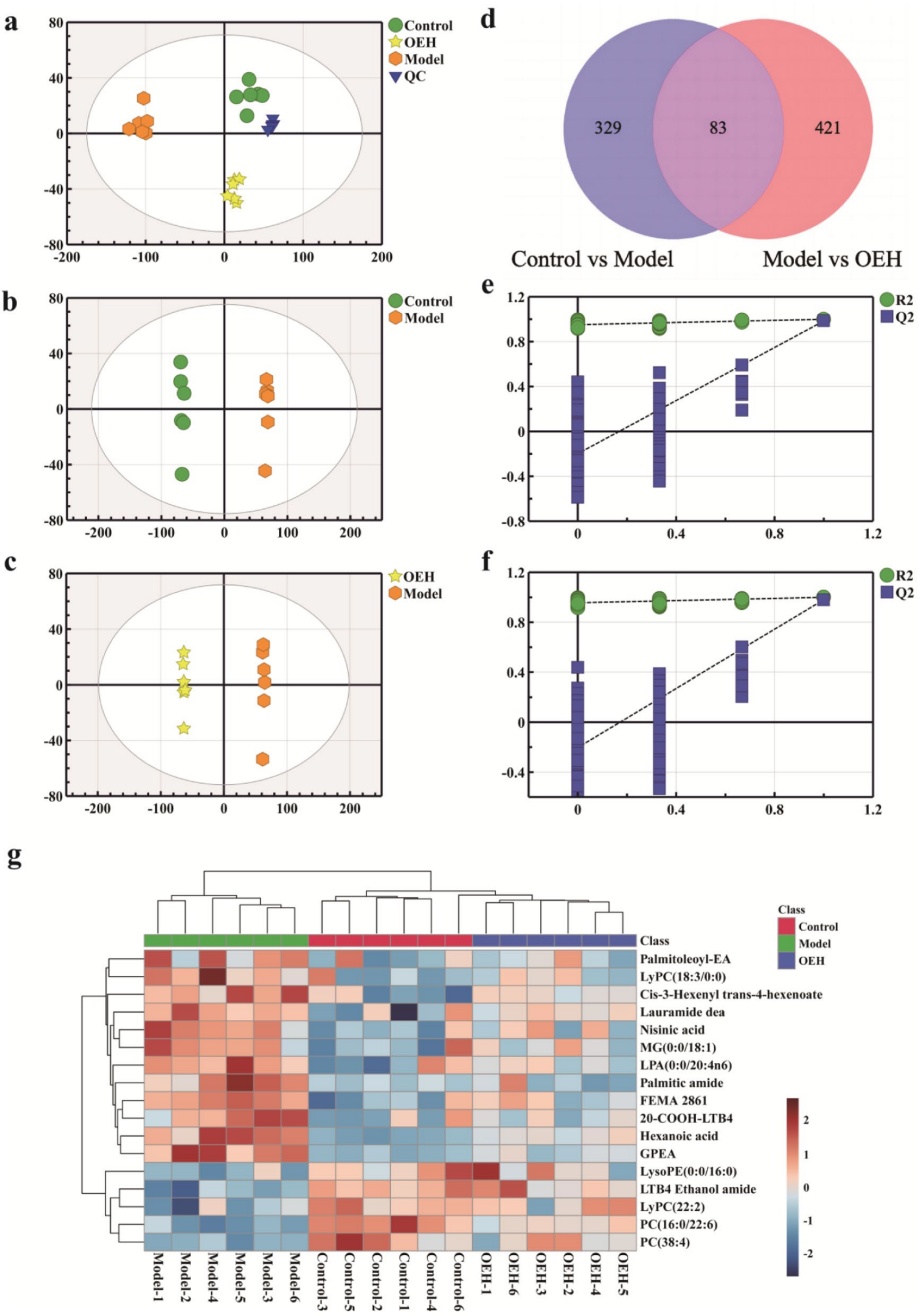
Lipidomics analysis and visualization heat map of OE on MD mice. **a** PCA score plot under total ion mode. **b** OPLS-DA plot between the blank group and the model group. **c** OPLS-DA plot between the model group and the OEH group. **d** Venn diagram of lipid metabolites among the blank group, the model group and the OEH group. **e** Score plot of the permutation test between the blank group and the model group. **f** Permutation test between the model group and the OEM group. **g** Visualization heat map

Serum metabolic profiling was performed in both positive and negative ion modes. The total ion current (TIC) chromatograms across different groups (Fig. 7) exhibited generally similar overall profiles but distinct mass spectrometry response intensities. Subsequently, principal component analysis (PCA) was applied to serum samples from the blank, model, and OEH groups. The PCA model (R^2^X = 0.551, Q^2^ = 0.453) demonstrated robustness in capturing metabolic variances, as evidenced by the clear separation among the three groups in both ion modes (Fig. 8a). This distinct clustering indicates significant intergroup differences in serum lipid metabolites.

Orthogonal partial least squares-discriminant analysis (OPLS-DA) further confirmed distinct separations in lipid metabolic profiles between the blank and model groups, as well as between the model and OEH groups, in both positive and negative ion modes (Fig. 8b–c, e–f). The high Q^2^ values (> 0.9) indicate an excellent model fit and predictive capability. To validate model robustness and guard against overfitting, a permutation test (*n* = 200) was conducted for each model. The results confirm that all OPLS-DA models are reliable and not overfitted.

Differential lipid metabolites were screened by applying a variable importance in the projection (VIP) threshold of > 1.0 from the OPLS-DA model and a statistical significance of *P* < 0.05. As shown in Fig. 8d, 83 common differential lipid metabolites were identified across the three groups. Following rigorous identification, 17 of these common metabolites were established as biomarkers through which OE ameliorates memory impairment (MD). Compared to the model group, the OEH group exhibited significant up-regulation of 4 serum lipid metabolites and significant down-regulation of 13.

Visual heatmap analysis revealed distinct expression patterns of metabolites between the blank and model groups. Following OEH treatment, these metabolite levels shifted significantly, normalizing toward the profile observed in the blank group (Fig. 8g). Subsequent cluster analysis of the identified differential lipid metabolites categorized them as follows: 9 fatty acids (FA), 7 glycerophospholipids (GP), and 1 glycerolipid (GL). This classification indicates that FAs represent the most abundant category of altered lipids, followed by GPs.

**Fig. 9:**
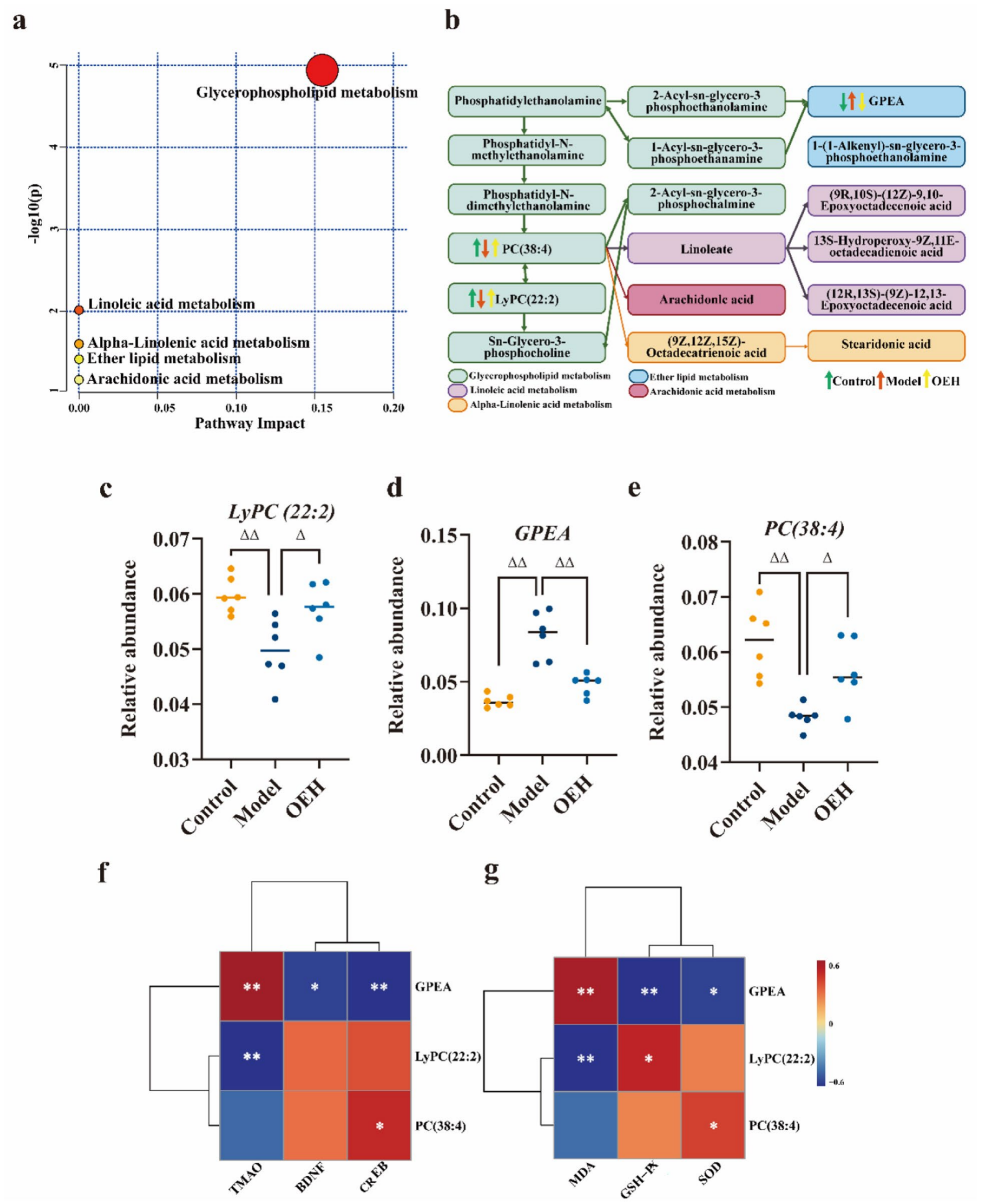
Metabolic pathway, visualization network diagram of metabolic pathway, differential metabolites of glycerophospholipids and correlation analysis. **a** Metabolic pathway. **b** Visualization network diagram of metabolic pathway. **c** Relative content of LyPC(22:2). **d** Relative content of GPEA. **e** Relative content of PC(38:4); *n* = 6, * x̄* ± *s*, Compared with the model group: ^∆^*P* < 0.05, ^∆∆^*P* < 0.01. **f** Correlation analysis between lipid metabolites and neuroprotection factors. **g** Correlation analysis between lipid metabolites and oxidative stress indexes; correlation strength: ^*^ P ≤ 0.05, ^**^ P ≤ 0.01

To elucidate the mechanisms underlying OE's ameliorative effect on MD, we performed metabolic pathway analysis on the 17 differential lipid metabolites. Enrichment analysis revealed five relevant pathways: glycerophospholipid metabolism, linoleic acid metabolism, α-linolenic acid metabolism, ether lipid metabolism, and arachidonic acid metabolism. Among these, glycerophospholipid metabolism emerged as the most significantly enriched pathway (Fig. 9a; Table 2).

**Table 2:** Metabolic pathway information

Metabolic pathway	Match status	*p*	− log(p)	Holm p	FDR	Impact
Glycerophospholipid metabolism	3/36	0.00001	4.93830	9.6822E-4	9.6822E-4	0.15471
Linoleic acid metabolism	1/5	0.00965	2.01540	0.80115	0.40540	0.00000
α-linolenic acid metabolism	1/13	0.02497	1.60260	1.00000	0.69907	0.00000
Ether lipid metabolism	1/20	0.03824	1.41750	1.00000	0.80297	0.00000
Arachidonic acid metabolism	1/36	0.06812	1.16680	1.00000	1.0000	0.00000

We subsequently integrated these findings to construct a metabolic network diagram delineating the regulatory effect of OE on MD (Fig. 9b). Arrows adjacent to the compounds indicate the direction of their content changes. Notably, PC(38:4) was implicated in four pathways: glycerophospholipid, linoleic acid, α-linolenic acid, and arachidonic acid metabolism. Similarly, LyPC(22:2) participated in glycerophospholipid metabolism, while GPEA was associated with both glycerophospholipid and ether lipid metabolism. The central role of these key metabolites underscores the critical importance of the glycerophospholipid metabolism pathway in mediating the therapeutic effects of OE against memory impairment.

Analysis of three key metabolites revealed significant alterations in LyPC(22:2), GPEA, and PC(38:4) within the glycerophospholipid metabolism pathway (Fig. 9c–e). Specifically, compared to the blank group, the model group exhibited significantly decreased serum levels of LyPC(22:2) (*P* < 0.01) and PC(38:4) (*P* < 0.01), alongside a significant increase in GPEA (*P* < 0.01). Treatment with OEH significantly reversed these alterations, elevating the levels of LyPC(22:2) (*P* < 0.05) and PC(38:4) (*P* < 0.05) while reducing GPEA (*P* < 0.01) compared to the model group. Collectively, these results demonstrate that polygala tenuifolia oligosaccharide esters ameliorate memory impairment by normalizing lipid metabolism dysregulation via the glycerophospholipid pathway.

The Spearman correlation was used to analyze the correlation coefficients between the three key lipid metabolites in the glycerophospholipid metabolism pathway and the neuroprotection indexes and oxidative stress indexes to determine the internal relationships among them.

As shown in Fig. 9f, the results of the correlation analysis with neuroprotection factors indicate that TMAO has a significantly positive correlation with GPEA (*P* < 0.01) and a significantly negative correlation with LyPC(22:2) (*P* < 0.01). BDNF has a significantly negative correlation with GPEA (*P* < 0.05). CREB has a significantly positive correlation with PC(38:4) (*P* < 0.05) and a significantly negative correlation with GPEA (*P* < 0.01).

As shown in Fig. 9g, the results of the correlation analysis with oxidative stress indexes indicate that MDA has a significantly positive correlation with GPEA (*P* < 0.01) and a significantly negative correlation with LyPC(22:2) (*P* < 0.01). GSH-PX has a significantly positive correlation with LyPC(22:2) (*P* < 0.05) and a significantly negative correlation with GPEA (*P* < 0.01). SOD has a significantly positive correlation with PC(38:4) (*P* < 0.05) and a significantly negative correlation with GPEA (*P* < 0.05).

The above results indicate that the expression levels of LyPC(22:2), PC(38:4), and GPEA in the glycerophospholipid pathway have significant correlations with neuroprotection factors and oxidative stress indexes, suggesting that OE may improve memory impairment by regulating glycerophospholipid metabolism.

### Discussing the mechanism of OE on MD mice based on the "gut-brain" axis theory

#### OE can repair colon tissue

**Fig. 10:**
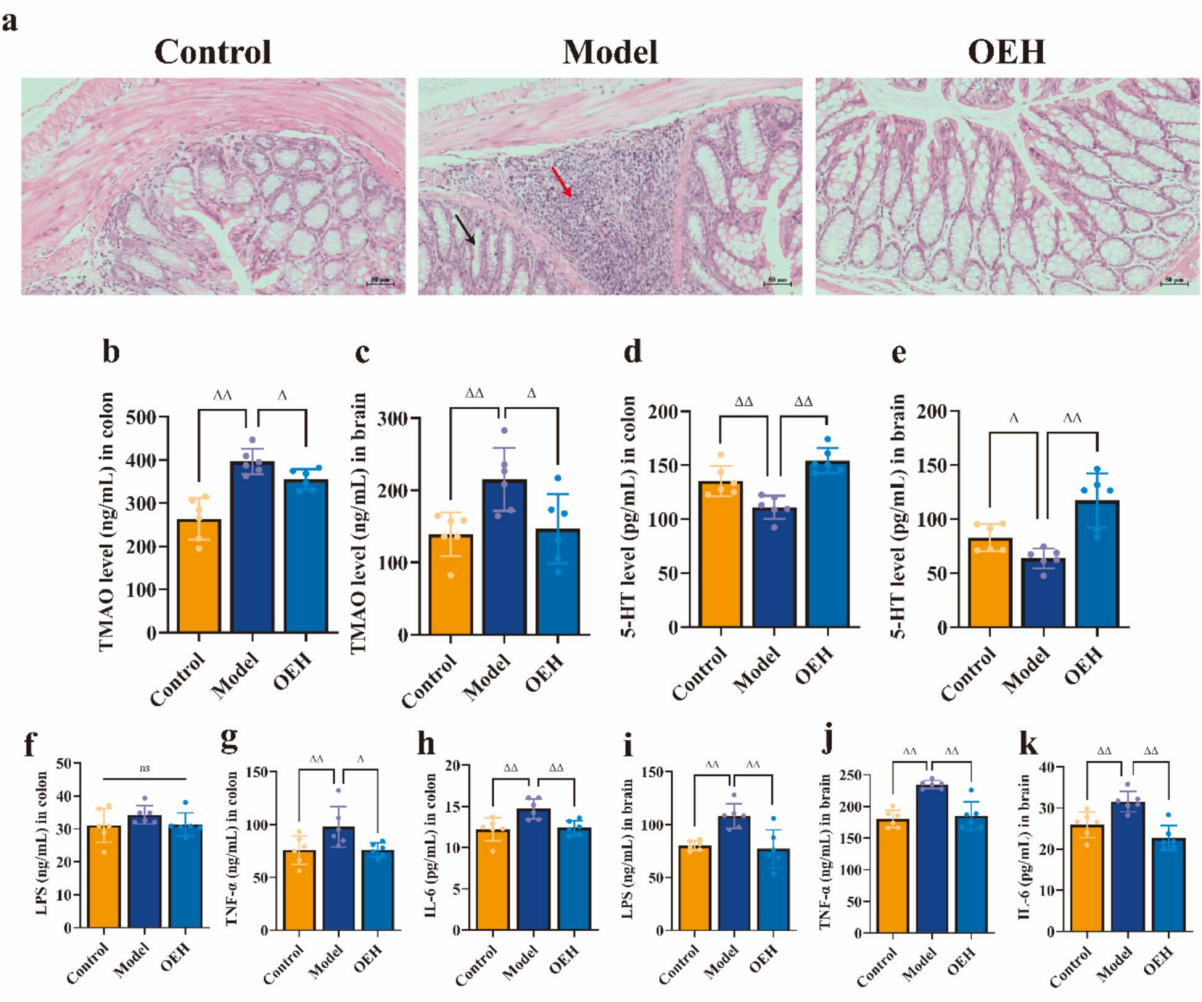
OE can repair colon tissue, reduce the levels of inflammatory factors and restore microbial metabolism. **a** Impact on mouse colon tissue (× 200). Black arrow: abnormal inflammatory infiltration—cryptitis; red arrow: abnormal inflammatory infiltration—increased stromal plasma cells. **b** TMAO levels in colon tissue. **c** TMAO levels in brain tissue. **d** 5-HT levels in colon tissue. **e** 5-HT levels in brain tissue; *n* = 6, *x̄* ± s, compared with the model group:^∆^*P* < 0.05,^∆∆^*P* < 0.01. **f**–**k** Contents of LPS, TNF-α, and IL-6 in mouse brain and colon tissues. **f**–**h** Brain tissue. **i**-**k** Colon tissue; *n* = 6, *x̄* ± s, compared with the model group:^∆^*P* < 0.05,^∆∆^*P* < 0.01, ns.: no significant difference

In the study, the colon mucosa of mice in the control group displayed intact epithelial structure. In contrast, the model group exhibited severe inflammation in the submucosa, with plasma cell infiltration causing edema, and neutrophil infiltration leading to crypt inflammation and a reduction in goblet cells. In the PT, OEL, OEM, and OEH groups, there was varying degrees of improvement in inflammation, evidenced by reduced inflammatory cell infiltration, restored glandular structure, and alleviated colon mucosal edema (Fig. 10a).

#### OE can restore microbial metabolite levels

To further elucidate the mechanism by which OE improves MD, we assessed the levels of TMAO and 5-HT in the brain and colon tissues of MD mice (Fig. 10b–e). In the brain tissue, TMAO levels in the model group were significantly higher compared to the control group (*P* < 0.01), while 5-HT levels were significantly lower (*P* < 0.05). After OEH treatment, TMAO levels in the brain tissue significantly decreased (*P* < 0.05), and 5-HT levels significantly increased (*P* < 0.01). Similarly, in colon tissue, TMAO levels were significantly elevated (*P* < 0.01) and 5-HT levels significantly reduced (*P* < 0.01) in the model group compared to the control. OEH treatment resulted in a significant decrease in TMAO levels (*P* < 0.05) and a significant increase in 5-HT levels (*P* < 0.01) in colon tissue. These findings indicate that OE administration can effectively regulate TMAO and 5-HT levels in both brain and colon tissues, consistent with the serum data. This suggests that MD may compromise the blood–brain barrier, allowing TMAO to enter the brain and affect brain function.

#### OE can reduce the levels of inflammatory factors

In brain tissue (Fig. 10f–h), the levels of LPS, TNF-α, and IL-6 in the model group significantly increased compared to the blank group (*P* < 0.01). In contrast, these levels significantly decreased in the OEH group compared to the model group (*P* < 0.01). In colon tissue (Fig. 10i–k), the levels of TNF-α and IL-6 in the model group significantly increased compared to the blank group (*P* < 0.01). Compared to the model group, the levels of TNF-α and IL-6 in the OEH group significantly decreased (*P* < 0.05, *P* < 0.01). Although the LPS content in the colon tissue of the model group significantly increased, it was downregulated after OE administration without a significant difference. These results suggest that gut microbiota dysbiosis may affect the permeability of the gut-brain barrier, leading to increased intestinal pro-inflammatory factors such as LPS, TNF-α, and IL-6. Polygala oligosaccharide esters can alleviate this phenomenon, further suggesting that MD may damage the gut-brain axis barrier.

#### Results of immunohistochemistry

**Fig. 11:**
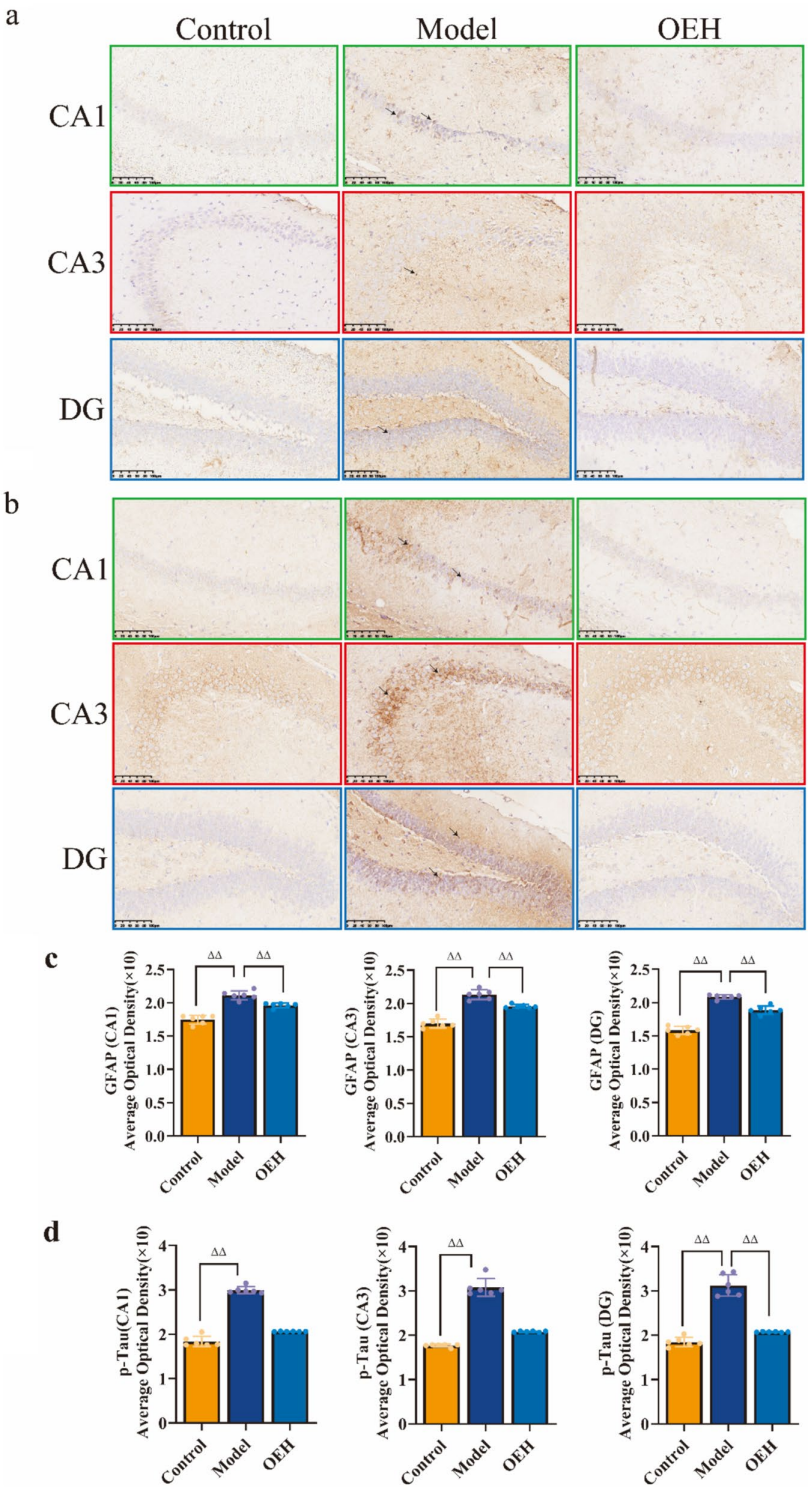
Results of immunohistochemistry for p-Tau and GFAP. **a** Results of immunohistochemistry for GFAP. **b** Expression levels of GFAP protein in CA1, CA3, and DG regions compared with the model group: ^*∆*^*P* < 0.05, ^∆∆^*P* < 0.01. **c** Results of immunohistochemistry for p-Tau. **d** Expression levels of p-Tau protein in CA1, CA3, and DG regions compared with the model group: ^∆^*P* < 0.05, ^∆∆^*P* < 0.01

As shown in Fig. 11, in the brain tissue, the levels of glial fibrillary acidic protein (GFAP) in the CA1, CA3, and DG regions of the model group were significantly higher than those of the blank group (*P* < 0.01). In contrast, the levels of GFAP in the CA1, CA3, and DG regions of the OEH group were significantly lower than those of the model group (*P* < 0.01). The levels of phosphorylated Tau protein (p-Tau) in the CA1, CA3, and DG regions of the model group were significantly higher than those of the blank group (P < 0.01). The level of p-Tau in the DG region of the OEH group was significantly lower than that of the model group (*P* < 0.01), and the levels of p-Tau in the CA1 and CA3 regions of the OEH group showed a decreasing trend compared with those of the model group.

#### Correlation analysis

To further elucidate the relationship between the “gut—brain” axis, we analyzed the levels of relevant factors in brain and colon tissues. Spearman correlation analysis was conducted with the top 10 relative abundance OTUs, LDA ≥ 4 phyla and genera (Firmicutes, Ligilactobacillus, HT002, and Muribaculaceae—unclassified), and the differential lipid metabolites (LyPC(22:2), PC(38:4) and GPEAF in the glycerophospholipid metabolic pathway to clarify their interaction relationships.

**Fig. 12:**
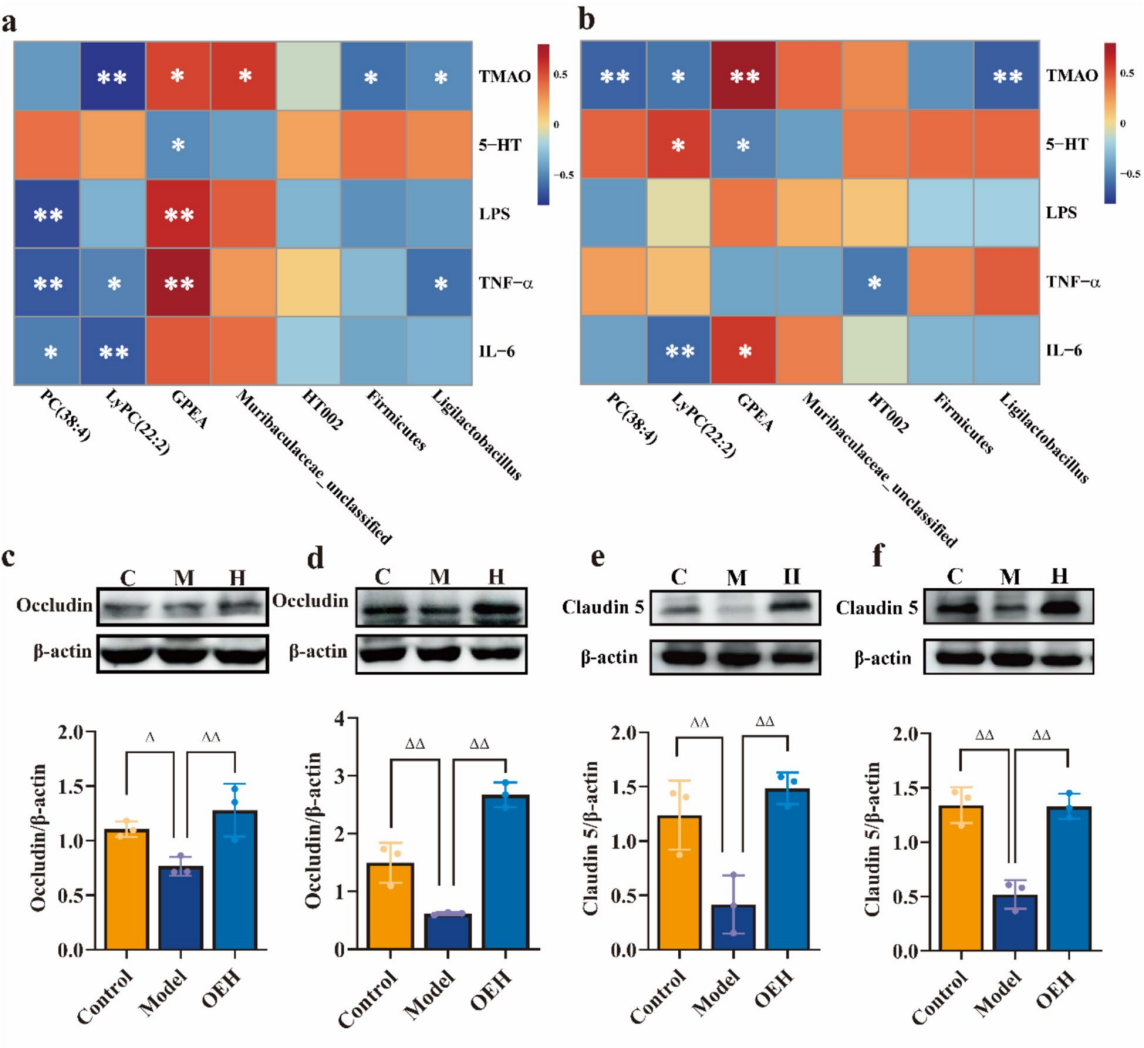
OE can improve the gut-brain barrier. **a** Correlation analysis between gut microbiota and brain tissue indicators. **b** Correlation analysis between gut microbiota and colon tissue indicators. **c**–**f** Expression of Occludin protein in brain and colon tissues. **c** Expression level of Occludin protein in brain tissue. **d** Expression level of Occludin protein in colon tissue; **e** expression level of Claudin 5 protein in brain tissue. **f** Expression level of Claudin 5 protein in colon tissue. *n* = 3, *x̄* ± s, compared with the model group: ^∆^*P* < 0.05, ^∆∆^*P* < 0.01

As shown in Fig. 12a, in brain tissue, TMAO was significantly positively correlated with Muribaculaceae-unclassified (*P* < 0.05) and GPEA (*P* < 0.05), and significantly negatively correlated with Ligilactobacillus (*P* < 0.05), Firmicutes (*P* < 0.05) and LyPC(22:2) (*P* < 0.01); 5-HT was significantly negatively correlated with GPEA (*P* < 0.05), and significantly positively correlated with HT002, Firmicutes, Ligilactobacillus, LyPC(22:2) and PC(38:4); LPS was significantly positively correlated with GPEA (*P* < 0.01) and significantly negatively correlated with PC(38:4) (*P* < 0.01); TNF-α was significantly positively correlated with GPEA (*P* < 0.01), and significantly negatively correlated with LyPC(22:2) (*P* < 0.05), PC(38:4) (*P* < 0.01) and Ligilactobacillus (*P* < 0.05); IL-6 was significantly negatively correlated with LyPC(22:2) (*P* < 0.01) and PC(38:4) (*P* < 0.05).

As shown in Fig. 12b, in colon tissue, TMAO was significantly positively correlated with GPEA (*P* < 0.01) and significantly negatively correlated with Ligilactobacillus (*P* < 0.01), LyPC(22:2) (*P* < 0.05) and PC(38:4) (*P* < 0.01); 5-HT was significantly positively correlated with LyPC(22:2) (*P* < 0.05) and significantly negatively correlated with GPEA (*P* < 0.05); TNF-α was significantly negatively correlated with HT002 (*P* < 0.05); IL-6 was significantly positively correlated with GPEA (*P* < 0.05) and significantly negatively correlated with LyPC(22:2) (*P* < 0.01).

#### OE can improve the gut-brain barrier

To further determine whether OE improve MD through the "gut-brain" axis, WB analysis was performed to assess the expression of key proteins, Occludin and Claudin 5, which are critical for the structure, permeability, and function of gut and brain tissues (Fig. 12c–f).

Compared to the blank group, the protein levels of Occludin (*P* < 0.05) and Claudin 5 (*P* < 0.01) were significantly reduced in the brain tissue of the model group. Similarly, in the colon tissue of the model group, the protein levels of Occludin (*P* < 0.01) and Claudin 5 (*P* < 0.01) were significantly decreased. In contrast, compared to the model group, the protein levels of Occludin (*P* < 0.05) and Claudin 5 (*P* < 0.01) were significantly increased in the brain tissue of the OEH group, and the protein levels of Occludin (*P* < 0.01) and Claudin 5 (*P* < 0.01) were also significantly increased in the colon tissue of the OEH group.

These results suggest that MD in mice can damage the gut and brain tissue barriers, facilitating the passage of TMAO, 5-HT, LPS, TNF-α, and IL-6 across the blood–brain barrier and exacerbating neuroinjury. Following OE treatment, significant repair of the gut and brain tissue barriers was observed, thus protecting the body's homeostasis.

### FMT treatment can enhance the learning and memory abilities of MD mice

**Fig. 13:**
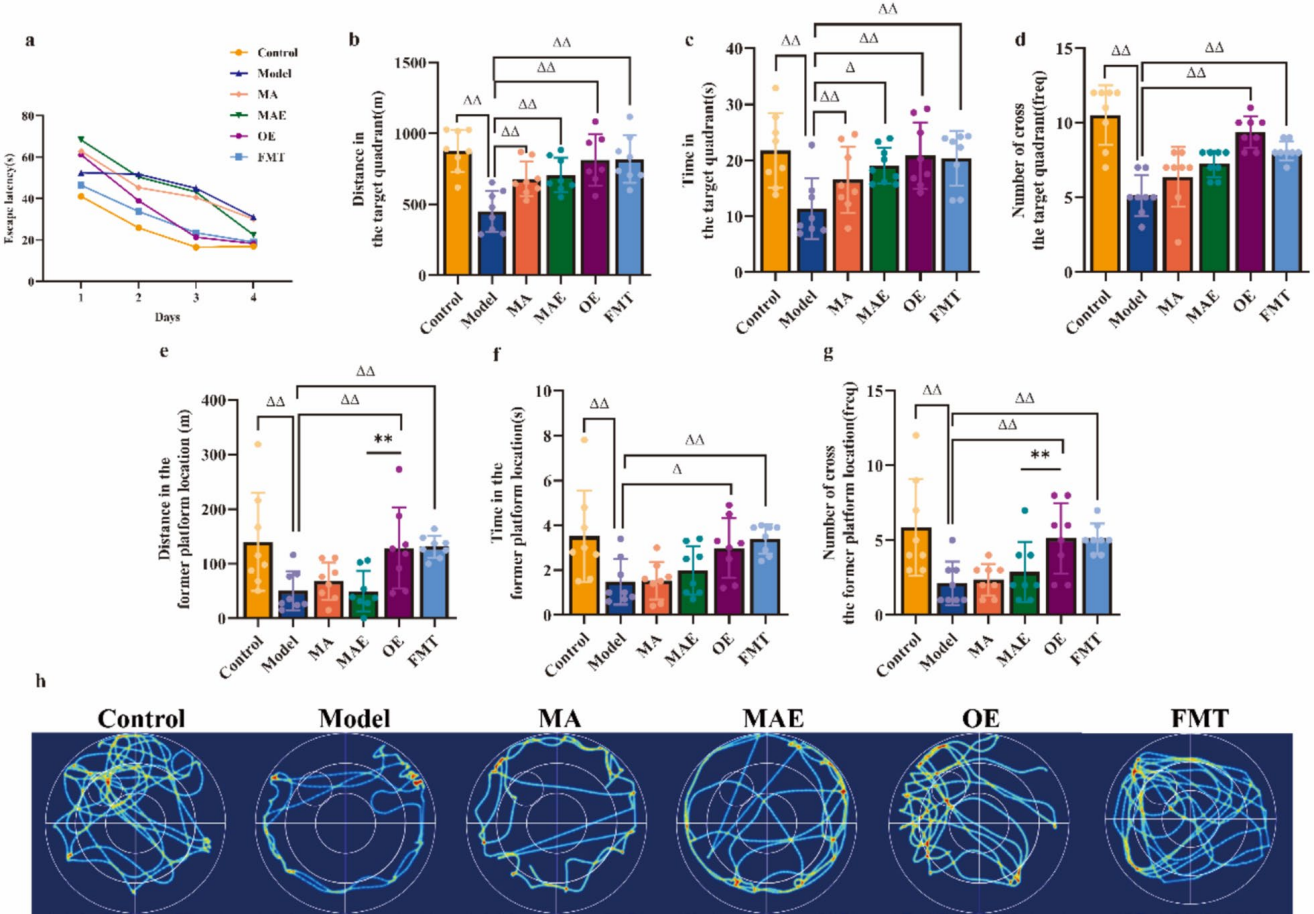
OE can enhance the learning and memory abilities of mice with memory disorder, repair brain tissue damage, and enhance neuroprotective effects. **a** Morris water maze spatial exploration test. **b** Distance moved in the target quadrant. **c** Time spent in the target quadrant. **d** Number of entries into the target quadrant. **e** Number of entries into the original platform. **f** Time spent in the original platform. **g** Distance moved in the original platform; *n* = 8, *x̄* ± s, compared with the model group, ^∆^*P* < 0.05, ^∆∆^*P* < 0.01;MAE group compared with OE group: ^*^P < 0.05, ^**^P < 0.01. **h** Track map of the spatial exploration test on the 5th day in the Morris water maze

In the MWM experiment of studying FMT in the treatment of MD mice, 4-day spatial navigation training was first carried out. The results showed that the escape latency of mice in each group decreased progressively (Fig. 13a). As shown in Fig. 13a, mice in the Control group displayed significantly shorter escape latencies at all training time points compared with those in the other groups. After 4-day spatial navigation training, the escape latencies of mice in all other groups showed a gradual decreasing trend, among which the OE group and FMT group exhibited the most significant reduction. These findings indicate that the modeling successfully induced spatial cognitive impairment in MD mice, and OE and FMT treatments exerted prominent ameliorative effects on the spatial cognitive impairment induced by modeling. In the spatial exploration test on the fifth day (Fig. 13b–g), compared with the Control group, the Distance in the target quadrant, Time in the target quadrant, Number of cross the target quadrant, Distance in the former platform location, Time in the former platform location and Number of cross the former platform location in the Model group were significantly decreased (*P* < 0.01). Compared with the Model group, the Distance in the target quadrant in the MA group was significantly increased (*P* < 0.01), the Distance in the target quadrant (*P* < 0.01) and Time in the target quadrant (*P* < 0.05) in the MAE group were significantly increased; the Distance in the target quadrant (*P* < 0.01), Time in the target quadrant (*P* < 0.01), Number of cross the target quadrant (*P* < 0.01), Distance in the former platform location (*P* < 0.01), Time in the former platform location (*P* < 0.05) and Number of cross the former platform location (*P* < 0.01) in the OE group were significantly increased, and the Distance in the target quadrant, Time in the target quadrant, Number of cross the target quadrant, Distance in the former platform location, Time in the former platform location and Number of cross the former platform location in the FMT treatment group were all significantly increased (*P* < 0.01). The above results indicate that FMT treatment improves spatial memory impairment and enhances learning and memory abilities in MD mice, and ablation of the gut microbiota partially diminished the cognitive-improving effects of OE.

### FMT treatment can repair brain tissue damage

**Fig. 14:**
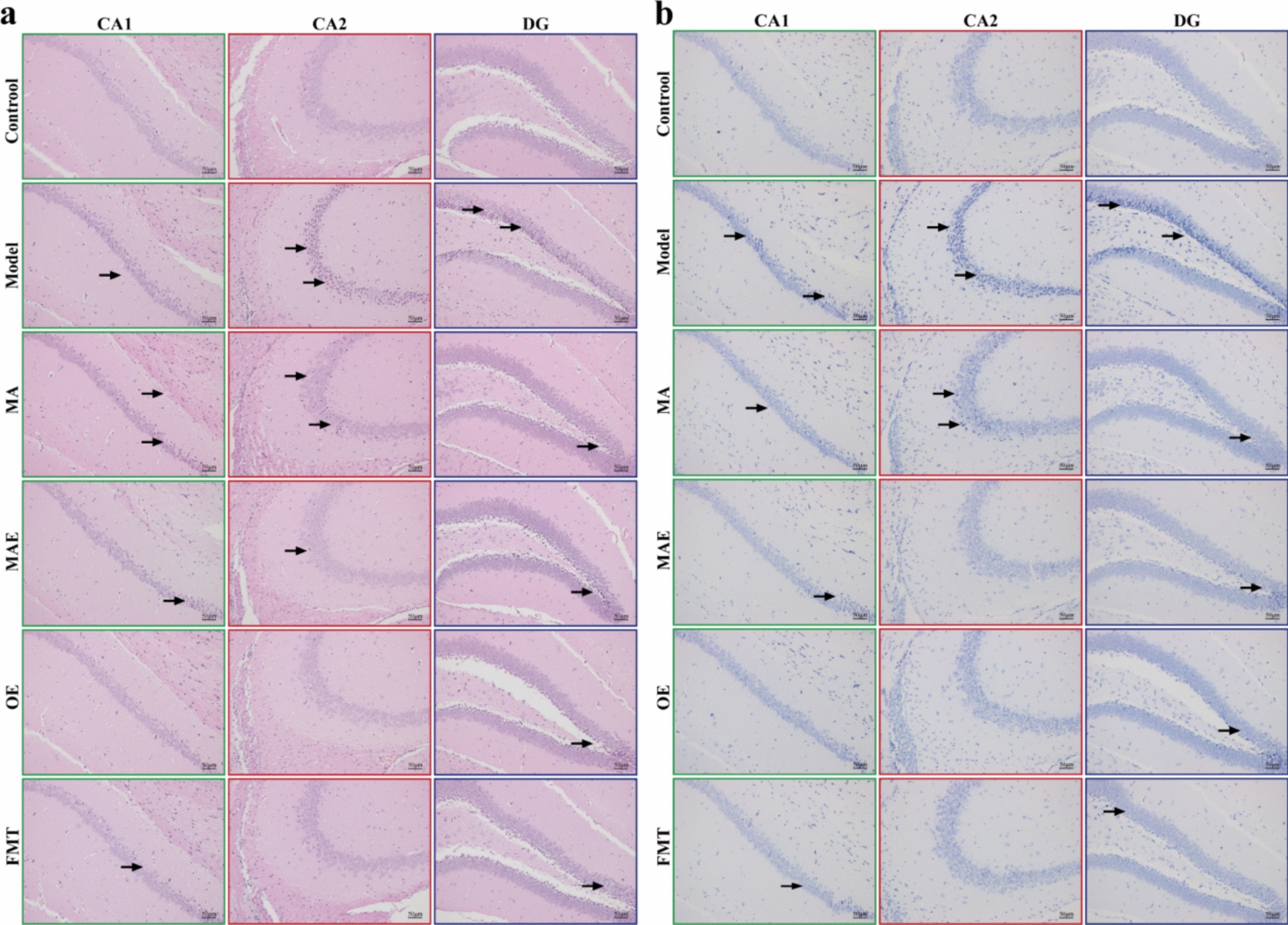
Fecal microbiota transplantation repairs brain tissue injury. **a** HE staining of mouse brain tissue (× 200). **b** Nissl staining of mouse brain tissue (× 200)

HE staining of brain tissue showed that in the control group, neuronal cells exhibited intact structure, tight arrangement, clear nuclear boundaries, normal cell size, large and round nuclei, and distinct nucleoli. In the model group, the structure of neuronal cells in the CA1, CA3, and DG regions was unclear, with disordered arrangement, irregular morphology, indistinct nuclear boundaries, reduced cell numbers, and obvious pyknosis. Compared with the model group, the OE and FMT groups exhibited regularly arranged neuronal cells, significantly increased cell numbers, and only minimal pyknosis, reflecting further recovery (Fig. 14a). Nissl staining of brain tissue revealed that in the control group, neuronal cells had intact structure and evenly distributed Nissl bodies. In the model group, neuronal cells in the CA1, CA3, and DG regions displayed irregular morphology, with Nissl bodies showing dissolution and substantial loss. Compared with the model group, the MA and MAE groups showed a gradual restoration of regular neuronal arrangement, relatively intact morphology, and reduced dissolution of Nissl bodies, with more marked improvement in the MAE group. Compared with the MAE group, the OE and FMT groups exhibited neatly arranged neuronal cells and a significantly increased number of Nissl bodies (Fig. 14b). Neuronal damage in the MA group was comparable to that observed in the Model group. Moreover, the amelioration of neuronal injury in the MAE group was less prominent than that in the OE group(Fig. 14a, b).

### FMT treatment can repair colon tissue in MD mice

**Fig. 15:**
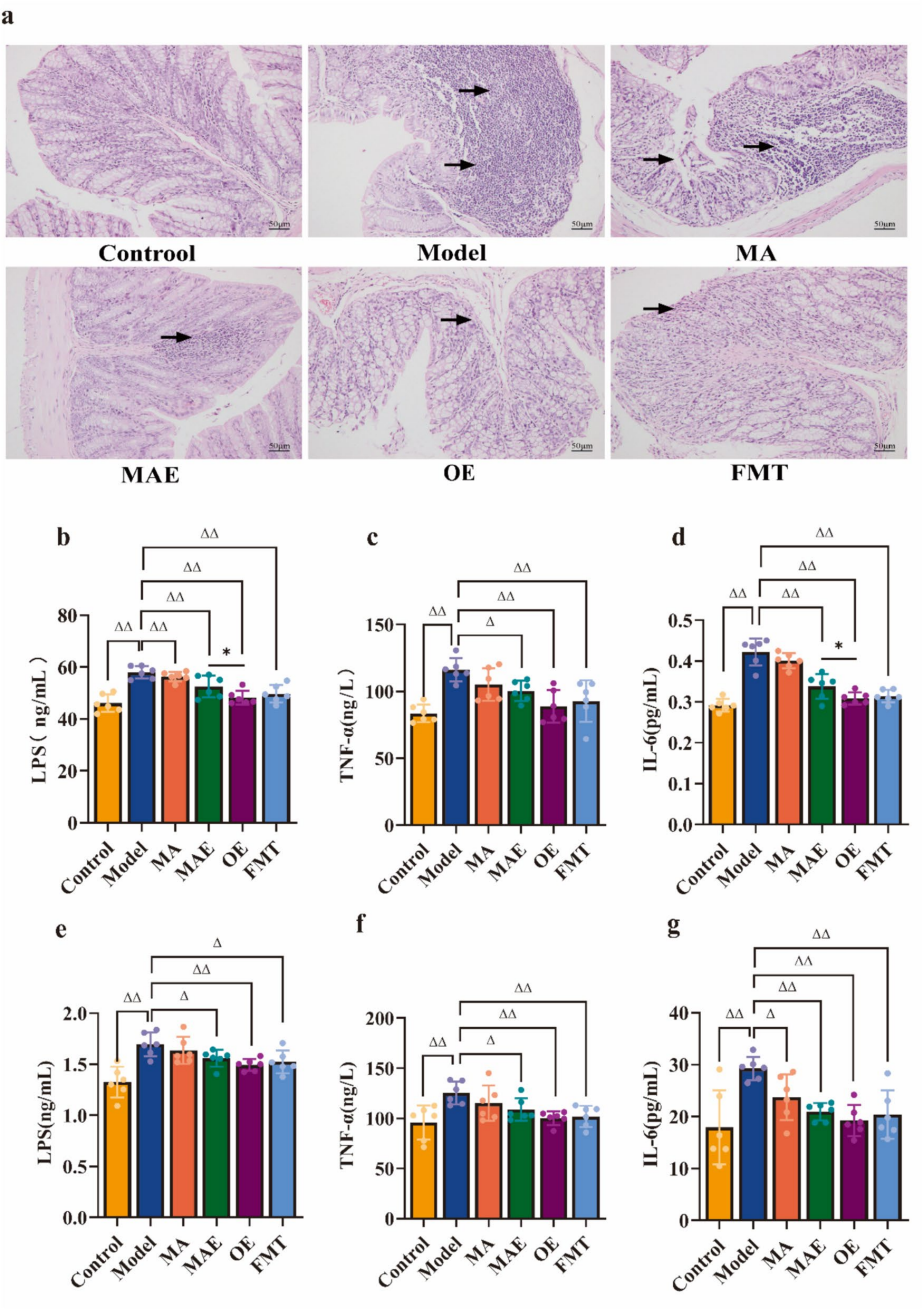
Fecal microbiota transplantation repaired colon tissue damage and reduced inflammatory factor levels in both brain and colon tissues. **a** HE staining of mouse colon tissue (× 200). **b**–**d** Levels of LPS, TNF-α, and IL-6 in mouse colon. **e**–**g** Levels of LPS, TNF-α, and IL-6 in mouse brain. *n* = 6, *x̄* ± s, compared with the model group: ^∆^*P* < 0.05,^∆∆^*P* < 0.01; MAE group compared with OE group: ^*^P < 0.05, ^**^P < 0.01

HE staining of colon tissue showed that in the control group, the colonic mucosa exhibited normal morphology, intact structure, normal crypts, and neatly arranged glands with essentially uniform shape and size, with no pathological changes observed. In the model group, severe epithelial cell shedding, extensive inflammatory cell infiltration, separation of the intestinal mucosal epithelium and lamina propria, disrupted and disordered tight junction structures between cells, decreased goblet cells, deformed and disorganized glands, and atrophy of intestinal glands and crypt loss were observed. Compared with the model group, the OE and FMT groups exhibited mild inflammatory infiltration in the lamina propria and submucosa, intact epithelial cell structure, and further amelioration of tissue damage. Compared with the OE group, the MAE group exhibited a reduction in goblet cell number, along with intestinal gland atrophy, crypt loss, and increased inflammatory infiltration (Fig. 15a).

### FMT treatment can alleviate inflammatory responses in the brain and colon tissues of MD mice

Compared with the control group, the levels of LPS, TNF-α, and IL-6 in colon tissue were significantly increased in the model group (*P* < 0.01). Compared with the model group, the MA group showed a significant decrease in LPS level (*P* < 0.01); the MAE group exhibited significant decreases in LPS and IL-6 levels (*P* < 0.01) and a significant decrease in TNF-α level (*P* < 0.05); the OE and MFT groups showed significant decreases in LPS, TNF-α, and IL-6 levels (*P* < 0.01). Compared with the MAE group, the OE group showed significant decreases in LPS and IL-6 levels (*P* < 0.05) (Fig. 15b–d).

Compared with the control group, the levels of LPS, TNF-α, and IL-6 in brain tissue were significantly increased in the model group (*P* < 0.01). Compared with the model group, the MA group showed a significant decrease in IL-6 level (*P* < 0.05); the MAE group exhibited a significant decrease in IL-6 level (*P* < 0.01) and significant decreases in LPS and TNF-α levels (*P* < 0.05); the OE group showed significant decreases in LPS, TNF-α, and IL-6 levels (*P* < 0.01); the MFT group exhibited significant decreases in TNF-α and IL-6 levels (*P* < 0.01) and a significant decrease in LPS level (*P* < 0.05) (Fig. 15e–g). These findings demonstrate that both OE and FMT treatments significantly alleviate inflammation in the colon and brain, whereas quadruple antibiotic administration markedly attenuates the therapeutic effect of OE on intestinal inflammation.

### FMT can reduce colonic permeability in MD mice

**Fig. 16:**
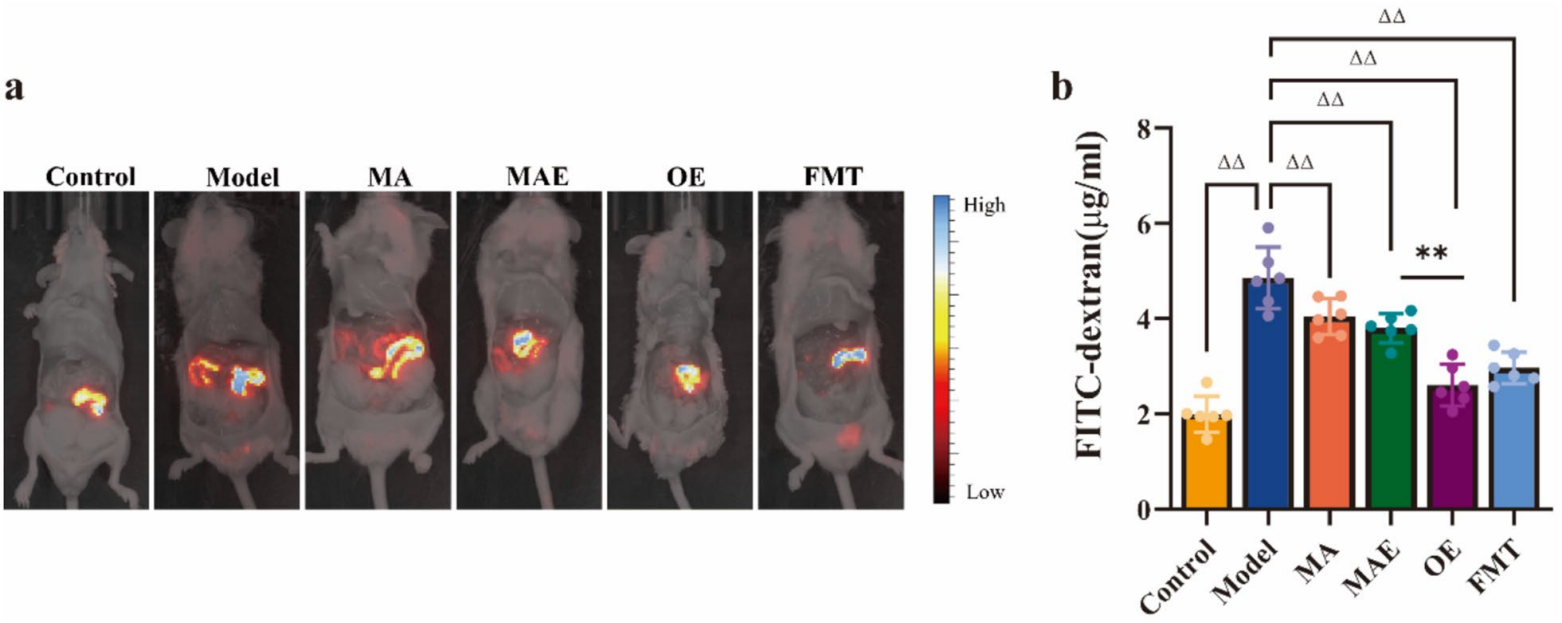
FMT reduced colonic permeability in MD mice. **a** HE staining of mouse colon tissue (× 200). **a** FITC-dextran distribution in the intestinal tract of mice. **b** Content of FITC-dextran in serum *n* = 6, *x̄* ± s. compared with the model group: ^∆^*P* < 0.05, ^∆∆^*P* < 0.01; MAE group compared with OE group: ^*^P < 0.05,^**^P < 0.01

The function and integrity of the intestinal barrier were assessed in the present study. In vivo fluorescence imaging demonstrated that 4 h following FD4 gavage, fluorescence signals in the control group were predominantly restricted to the colon. In contrast, marked extravasation and diffusion of fluorescence into the abdominal cavity were observed in the Model, MA, and MAE groups, whereas such diffusion was negligible in the OE and FMT groups (Fig. 16a). These observations indicate that modeling successfully disrupted intestinal barrier function.

Quantitative measurement of serum FD4 fluorescence intensity yielded results consistent with the imaging findings. Relative to the Control group, serum FD4 levels were significantly elevated in the Model group (*P* < 0.01), and this increase was significantly reversed by both OE and FMT interventions (*P* < 0.01) (Fig. 16b).

Furthermore, serum FITC-dextran fluorescence intensity was significantly lower in the OE group than in the MAE group (*P* < 0.01), implying that the therapeutic efficacy of OE was markedly attenuated following gut microbiota depletion with quadruple antibiotics. Collectively, these results confirm that OE exerts its pharmacological effects via modulation of the gut microbiota in MD model mice.

## Discussion

MD is a common clinical condition with an unclear etiology and mechanism. Studies have indicated a link between the abnormal accumulation of senile plaques and neurofibrillary tangles in neuropathological mechanisms, which promote free radical damage, reduce cholinergic activity, and lead to memory and cognitive dysfunction [19]. Existing clinical treatments can only temporarily alleviate symptoms and do not prevent the degenerative progression of the disease or restore lost neurons. Given the multiple pathological factors involved in MD, single-target drugs are often inadequate for addressing all pathogenic factors. Thus, multi-target drugs offer a promising strategy for potential prevention or slowing of disease progression [20, 21].

Polygala oligosaccharide esters, a component of Polygala, exhibit significant neuroprotective and antidepressant properties. Modern studies have shown that various components of OE possess pharmacological activities, such as enhancing learning and memory, protecting neurons, and improving synaptic plasticity [22]. Effective animal models are crucial for studying disease mechanisms and drug actions. The MD mouse model induced by d-galactose combined with aluminum chloride displays pathological changes, including neuronal apoptosis, cholinergic system damage, and OS. Research indicates that this model affects neurotransmitter release in the brain and disrupts the gut microbiota [23]. Thus, this MD mouse model can be utilized to explore the mechanisms of MD-related drugs by regulating the gut microbiota. This study used the Morris water maze (MWM) and other behavioral experiments, classic tools for assessing animal learning, memory, dementia, and aging [24], to evaluate OE’s effects on the learning and memory abilities of MD mice. The MWM results showed that both the OEM and OEH groups increased stay time, movement distance, and entries into the target quadrant and original platform in MD mice, indicating that OE effectively improves MD. To further assess whether OE protects neurons in brain tissue, we performed Nissl staining. The staining results confirmed OE's protective effect on hippocampal neurons in MD mice, evidenced by orderly cell arrangement, clear nuclei, and increased Nissl bodies in the CA1, CA3, and DG regions compared to the model group, highlighting its therapeutic potential in alleviating MD. CREB is a key transcription factor that regulates BDNF and memory formation [25]. BDNF binds to the neurotrophin receptor tyrosine kinase receptor B (TRKB), inducing TRKB dimerization and autophosphorylation [26], which activates downstream extracellular regulated protein kinases (ERK) and serine/threonine kinase 1 (AKT1) [27], promoting CREB phosphorylation. This positive feedback enhances BDNF, supports synaptic plasticity, neurogenesis, and reduces neuronal apoptosis [28]. Previous studies have confirmed that CREB-regulated BDNF levels are significantly decreased in the brains of MD patients [25]. OE significantly increased CREB and BDNF levels in the serum of MD mice, enhancing their learning and memory abilities, with the OEH group showing the most significant pharmacological efficacy.

Studies have shown that the gut microbiota in healthy individuals remains stable due to the mutual regulation among various bacterial groups. However, under certain pathological conditions, the intestinal microenvironment may promote the overgrowth of specific bacterial groups, which may further lead to the dysregulation of gut microbiota homeostasis. Then, it can affect brain function through the microbiota-gut-brain axis, causing behavioral and psychiatric disorders in the body [29]. 16S rDNA sequencing results show that OTU (Operational Taxonomic Units) clusters differ among groups, with a significant increase in OTU numbers in the model group. Compared to the model group, the OEH group exhibits a reduction in OTU numbers. Analysis of the α diversity of gut microbiota shows that the α diversity in the model group is higher than in the blank group. After OEH treatment, the α diversity of MD mice is adjusted to match that of the blank group. Similarly, β diversity analysis reflects differences in the composition or structure of gut microbiota among groups. A significant difference is observed between the control group and the model group, with OEH administration balancing the imbalance in the gut microbiota structure of MD mice. Firmicutes and Bacteroidetes are important phyla of human gut bacteria.

Imbalances in Firmicutes and Bacteroidetes are often observed in various brain diseases, such as neurodegenerative diseases, stroke, and hypertension [30]. In conditions of cognitive impairment, gut microbiota diversity in feces decreases, with a significant reduction in Firmicutes abundance and an increase in Bacteroidetes abundance, leading to a decreased ratio of Firmicutes to Bacteroidetes [8]. Research shows that people with higher gut microbiota diversity perform better in cognitive tests such as memory, and Lachnospiraceae is one of the key microbial groups related to cognitive abilities [31]. The results of this study indicate a significant change in the species composition of the gut microbiota in MD mice. Analysis at the phylum level reveals that the relative abundance of Firmicutes in MD mice is significantly reduced, while the relative abundance of Bacteroidetes is significantly increased. OE can restore this microbiota imbalance by regulating the Firmicutes-to-Bacteroidetes ratio. Linear discriminant analysis shows that Firmicutes is a characteristic phylum in the blank group. The OEH group significantly increases the relative abundance of Lachnospiraceae_NK4A136_group and Clostridiales_unclassified within Firmicutes, thereby restoring microbial balance. Through the functional prediction analysis of PICRUSt2, it is clear that OE can improve the activities of pathways such as the Nervous System, Excretory System, Cell Growth and Death, Folding, Sorting and Degradation, Amino Acid Metabolism, Energy Metabolism, and Metabolic Diseases. It has a particularly obvious effect on pathways such as Alzheimer's disease and lipid metabolism. The above results indicate that OE can improve memory impairment and restore the composition and relative abundance of key gut microbiota. Meanwhile, they also suggest that OE may exert its ameliorative effects on memory impairment by regulating lipid metabolism. Abnormal lipid metabolism is one of the earliest proposed pathogenic mechanisms of AD. The results of multiple studies have shown that abnormal lipid metabolism can induce the hyperphosphorylation and aggregation of tau protein, which further leads to downstream enzymatic reactions and ultimately affects the pathological process of AD [32, 33].

Lipids are the main macromolecular components in the brain [34]. During the regeneration process of the Central Nervous System (CNS), adipose-derived stem cells (Adult stem cells, ASC) produce a variety of neurotrophic factors, which can enhance the growth of neural synapses and provide neuroprotective effects for them [35, 36]. Phosphatidic acid (PA) is one type of Fatty Acids (FA) and has neurotoxicity [37]. An increase in PA will lead to the activation of microglia and astrocytes, oxidative stress, etc., causing cognitive decline, excessive inflammation, and brain atrophy [38, 39]. Aging and neurodegenerative diseases can cause microglia to become inflamed and secrete Leukotrienes (LTs). Leukotriene B4 (LTB4), as one type of LTs, is generated from Arachidonic acid (AA). Previous studies have shown that LTB4 plays a crucial regulatory role in inflammatory pathological processes such as nerve function damage and blood–brain barrier disruption [40, 41]. Lysophosphatidylcholine (LPC) can enhance neurotoxicity [42]. The level of LPC will increase in patients with repetitive mild traumatic brain injury, while it decreases in the plasma of patients with Alzheimer's disease [43–45]. In this study, a total of 17 differential biomarkers closely related to memory impairment were identified using the UPLC-MS mass spectrometry system (see Table 3 at the end of the article for details). The results showed that after treatment with OE, it was able to regulate the changes in the contents of various lipid metabolites caused by memory impairment. The changes mainly occurred in three types of lipid metabolites, namely Fatty Acids (FA), Glycerophosphates (GP), and Glycerolipids (GL). This result is consistent with the findings from the analysis of the 3rd-level KEGG metabolic pathways of the gut microbiota part, which indicated that OE can restore the activity of the lipid metabolism pathway and inhibit the activity of the Alzheimer's disease metabolic pathway. In this study, the content of 20-COOH-LTB4 in the serum of the mice in the model group was significantly higher than that in the blank group, and it decreased significantly after treatment with OE. This suggests that OE may improve memory impairment by regulating the content of LTB4. Therefore, LTB4 may be a key target for OE to improve memory impairment. In this study, the level of LyPC(22:2) increased significantly in the blank group, while the level of LyPC(18:3/0:0) decreased significantly in the blank group. This trend was exactly the opposite of that in the model group. After treatment with OE, the levels of these two differential lipid metabolites significantly returned to normal, indicating that the LPC levels show different trends in different animal models of diseases.

**Table 3:** Seventeen differential lipid metabolites in the serum of mice

No.	Common name	Formula	M/Z	Rt(min)	Reference ion	HMDB-ID	Main class	DeltaMass (ppm)	Control/model	OEH/model
1	Palmitic amide	C16H33NO	256.26352	12.79	[M + H] + 1	HMDB0012273	FA	0.14	↓∆∆	↓∆
2	FEMA 2861	C12H16O2	191.10699	8.269	[M − H] − 1	HMDB0035014	FA	-2.61	↓∆∆	↓∆∆
3	Hexanoic acid	C13H26NO4	259.15433	8.005	[M + H] + 1	HMDB0000756	FA	0.73	↓∆∆	↓∆∆
4	20-COOH-LTB4	C20H30O6	367.20959	8.449	[M + H] + 1	HMDB0006059	FA	4.88	↓∆	↓∆
5	Lauramide dea	C16H33NO3	288.25341	8.906	[M + H] + 1	HMDB0032358	FA	0.31	↓∆	↓∆
6	Cis-3-Hexenyl trans-4-hexenoate	C12H20O2	197.15396	7.424	[M + H] + 1	HMDB0031692	FA	1.51	↓∆∆	↓∆
7	LTB4 Ethanol amide	C22H37NO4	379.30589	7.391	[M + H] + 1	HMDB0002304	FA	4.84	↑∆∆	↑∆∆
8	Palmitoleoyl-EA	C18H35NO2	297.24379	13.935	[M − H] − 1	HMDB0013648	FA	0.91	↓∆	↓∆
9	Nisinic acid	C24H36O2	356.27966	8.500	[M + H] + 1	HMDB0002007	FA	-3.41	↓∆∆	↓∆
10	MG(0:0/18:1)	C21H40O4	356.28002	8.485	[M + H] + 1	HMDB0011537	GL	-6.22	↓∆	↓∆
11	LyPC(22:2)	C30H58NO7P	575.38124	6.747	[M + H] + 1	HMDB0010400	GP	-3.96	↑∆∆	↑∆
12	LPA(0:0/20:4n6)	C23H39O7P	457.23687	15.269	[M − H] − 1	HMDB0012496	GP	-1.96	↓∆	↓∆∆
13	PC(16:0/22:6)	C46H82NO7P	396.80194	5.822	[M + 2H] + 2	HMDB0013409	GP	1.09	↓∆∆	↓∆∆
14	GPEA	C5H14NO6P	216.06367	0.815	[M + H] + 1	HMDB0000114	GP	2.43	↓∆∆	↓∆∆
15	PC(38:4)	C46H84NO8P	405.80723	5.331	[M + 2H] + 2	HMDB0007988	GP	1.07	↑∆∆	↑∆
16	LyPC(18:3/0:0)	C26H48NO7P	517.31796	8.793	[M − H] − 1	HMDB0010388	GP	5.34	↓∆	↓∆
17	LysoPE(0:0/16:0)	C21H44NO7P	452.27893	9.884	[M − H] − 1	HMDB0011473	GP	1.61	↑∆∆	↑∆

*FA* Fatty Acids, *GP* Glycerophospholipids, *GL* Monoradylglycerols, ↑ up, ↓ down, 与模型组比: *n* = 6, ^∆^*P* < 0.05, ^∆∆^*P* < 0.01

Glycerophospholipids are involved in important processes such as the formation of biological membranes and signal transduction. Phosphatidylcholine synthesized from them is a key neurotransmitter. Studies have shown that supplementing Glycerophosphocholine (GPC) can delay the aging of the mouse brain, reduce the deposition of Transthyretin (TTR), and inhibit neuroinflammation [46]. Under the stimulation of chronic inflammation, changes in the levels of Neurotrophic factors (NTFs) can lead to memory dysfunction [47, 48]. In the state of oxidative stress in the body, it is likely to cause an increase in the number of Reactive oxygen species (ROS), induce Lipid peroxidation (LPO) of the phospholipids and cholesterol esters of Polyunsaturated fatty acid (PUFA) in the cell membrane and lipoprotein, generate lipid oxidation metabolites, and lead to a decrease in the activities of Superoxide Dismutase (SOD) and Glutathione (GSH) and an increase in the activity of Malondialdehyde (MDA) in the body's antioxidant system. In this study, the Metaboanalyst 5.0 metabolic pathway analysis was used to find that the oligosaccharide esters of Polygala tenuifolia can improve memory impairment through five pathways, including glycerophospholipid metabolism and linoleic acid metabolism, among which glycerophospholipid metabolism may play a major role. The levels of differential metabolites LyPC(22:2), PC(38:4), and GPEA in the glycerophospholipid metabolism pathway have changed. Compared with the blank group, the levels of LyPC(22:2) and PC(38:4) in the model group decreased significantly, and the level of GPEA increased significantly. After treatment with the oligosaccharide esters of Polygala tenuifolia, the levels of these three lipid metabolites significantly returned to normal. The above results suggest that Polygala tenuifolia oligosaccharide esters can ameliorate D-galactose/AlCl₃-induced memory impairment by regulating the homeostasis of glycerophospholipid metabolism, and its effect may be associated with modulating the metabolic balance of phosphatidylcholine and lysophosphatidylcholine. The Spearman correlation analysis also confirmed that the changes in the levels of these metabolites are consistent with the expression trends of the neuroprotective factors TMAO, CREB, and BDNF, and also consistent with the activities of the oxidative stress indicators MDA, SOD, and GSH-PX. The oligosaccharide esters of Polygala tenuifolia can improve cognitive function in mice with memory impairment by regulating metabolic pathways such as glycerophospholipid metabolism. Its mechanism of action may be associated with modulating lipid metabolic homeostasis, enhancing the body's anti-oxidative stress capacity, and exerting neuroprotective effects. Therefore, based on the above research results, it is shown that the oligosaccharide esters of Polygala tenuifolia can enhance the neuroprotective and anti-oxidative stress abilities of mice with memory impairment by regulating the glycerophospholipid metabolism pathway.

Dysbiosis of the gut microbiota, the release of intestinal pro-inflammatory factors, and increased intestinal barrier permeability form a tightly interlinked, self-reinforcing pathological cycle [49]. Disruption of microbial homeostasis directly impairs intestinal barrier integrity through multiple mechanisms: toxic microbial metabolites (e.g., LPS) attack and degrade key tight junction proteins (e.g., Occludin, Claudin, ZO-1), compromising the physical barrier, while simultaneously diminishing the thickness and density of the protective mucus layer, rendering the epithelium more vulnerable [50]. Crucially, dysbiosis potently activates local intestinal immunity-microbial components (e.g., LPS, flagellin), acting as pathogen-associated molecular patterns (PAMPs), are recognized by pattern recognition receptors (e.g., TLRs, NLRs) on epithelial cells and lamina propria immune cells [51, 52]. This recognition activates core inflammatory signaling pathways (e.g., NF-κB, MAPK), leading to the explosive release of abundant pro-inflammatory cytokines (e.g., TNF-α, IL-1β, IL-6, IFN-γ) [53].

These induced intestinal pro-inflammatory factors exert significant direct damaging effects on the gut barrier, further disrupting its structure and markedly increasing intestinal epithelial permeability. This heightened permeability represents a critical turning point in the escalating cycle, enabling the translocation of luminal-restricted substances-including microbiota-derived metabolites (e.g., LPS, SCFAs, bacterial DNA, peptidoglycan) and locally produced pro-inflammatory factors-into systemic circulation [54]. Circulating inflammatory mediators, particularly LPS and pro-inflammatory cytokines, reach the central nervous system via the bloodstream [55]. They can impair blood–brain barrier function and, more significantly, activate resident brain immune cells (microglia and astrocytes), triggering a neuroinflammatory cascade that releases additional brain-derived pro-inflammatory factors [56]. Critically, this neuroinflammation ultimately disrupts normal neural function and is implicated in the pathogenesis of anxiety, depression, cognitive impairment, and various neurodegenerative diseases [57, 58]. Furthermore, the brain, upon sensing stress or inflammatory signals, activates the hypothalamic–pituitary–adrenal (HPA) axis and autonomic nervous system (notably the sympathetic branch), releasing stress hormones (e.g., cortisol) and neurotransmitters (e.g., 5-HT, NE) [59, 60]. These signals feedback onto the gut, influencing its motility, secretion, blood flow, immune function, and microbiota composition, thereby further exacerbating intestinal inflammation, dysbiosis, and barrier damage, completing and sustaining this gut-brain pathological loop [61, 62].

TMAO (trimethylamine N-oxide) can induce gut microbiota imbalance, trigger neuroinflammation, and promote the accumulation of β-amyloid protein and tau protein [63, 64]. Researchers have detected TMAO in the cerebrospinal fluid of AD (Alzheimer's disease) patients, indicating that TMAO may enter the brains of AD patients through the Blood–brain barrier (BBB) [23]. 5-HT (serotonin) is one of the important indoleamine neurotransmitters linking the "gut-brain" axis. The disorder of the 5-hydroxytryptamine neurotransmitter system may lead to symptoms such as emotional and cognitive dysfunction, and metabolic disorders [65]. Microglia can maintain brain homeostasis. In pathological conditions, they will release pro-inflammatory factors (such as IL-6 and TNF-α), which in turn damage neurons and cause learning and memory dysfunction. Lipopolysaccharide (LPS) is present in the hippocampus and the neocortex of the temporal lobe of AD patients, and it has extremely strong pro-inflammatory ability [66, 67]. As AD patients age, intestinal function and the levels of pathogenic bacteria decline, resulting in a decrease in the levels of anti-inflammatory bacteria in the gut and an increase in the production of LPS, which further causes chronic inflammation in the intestine. Meanwhile, studies have also shown that LPS can increase the permeability of the intestine, disrupt the blood–brain barrier, activate microglia in the brain, and increase the release of IL-6 and TNF-α, leading to inflammation in the nervous system and accelerating the development process of neurodegenerative diseases [68]. Claudin and Occludin can regulate the permeability of the barrier and play a crucial role in maintaining the normal physiological functions of the intestinal barrier and the blood–brain barrier [69]. The hyperphosphorylation of Tau protein within neurons forms p-Tau, leading to the formation of neurofibrillary tangles, which can cause neuronal dysfunction and death. The damage to neurons will activate astrocytes, causing them to proliferate massively and upregulate the expression of GFAP [70].

To further confirm that OE can improve memory impairment (MD) by regulating the gut-brain axis, we detected the levels of TMAO, 5-HT, LPS, TNF-α, and IL-6 in brain and colon tissues, and performed Spearman correlation analysis between these substances, gut microbiota, and glycerophospholipid metabolites. Subsequently, we verified the permeability of the gut-brain barrier by detecting the protein levels of Occludin and Claudin-5. The research results showed that after induction with D-galactose combined with aluminum chloride, the levels of TMAO in the serum, brain, and colon of MD mice increased significantly, suggesting that memory impairment may affect the disorder of gut microbiota, leading to an increase in the level of TMAO and a decrease in the content of 5-HT in the gut. It further passes through the blood–brain barrier, causing nerve damage in the brain and aggravating memory impairment. After treatment with the oligosaccharide esters of OE, the content of TMAO can be significantly reduced, the level of 5-HT can be increased, and the damage of memory impairment can be alleviated. The levels of TNF-α and IL-6 in the brain and colon tissues of MD mice increased significantly, while OE can significantly reduce the levels of TNF-α and IL-6. The significant increase of LPS in the brain and colon tissues indicates an increase in intestinal permeability. This result suggests that the combination of D-galactose and aluminum chloride may lead to an increase in intestinal permeability. After administration of OE, the content of LPS decreased, indicating that OE can reduce microbiota-derived LPS and decrease the intestinal permeability of mice. At the same time, the inflammatory response and oxidative stress always coexist in diseases, and they promote and accelerate the development of diseases mutually, which further proves the anti-oxidative stress effect of OE. In addition, the results of the Spearman correlation analysis showed that under pathological conditions, the changes in the abundances of Firmicutes, Ligilactobacillus, and unclassified Muribaculaceae in the gut microbiota have a significant impact on memory impairment, which may lead to the expression of related factors in the brain and colon tissues and cause abnormal lipid metabolism. OE can improve MD by regulating the relative abundances of these microbiota and influencing the levels of TMAO, 5-HT, LPS, TNF-α, and IL-6 in the brain and colon tissues. This indicates that OE can improve MD by stabilizing the gut microbiota. The level of TMAO in brain tissue shows a negative correlation pattern, which suggests that under pathological conditions, changes in the abundances of Firmicutes, Ligilactobacillus, and unclassified Muribaculaceae have a significant impact on MD. These changes may affect the concentrations of 5-HT and TMAO in the brain and colon tissues. Significant alterations in the gut microbial metabolite TMAO and the neurotransmitter 5-HT may lead to MD, causing ecological imbalances of Ligilactobacillus, Firmicutes, and unclassified Muribaculaceae, which in turn result in damage to the intestinal mucosa. These disruptions can cross the blood–brain barrier, causing neuronal damage and exacerbating MD. The results of Western Blot (WB) showed that the levels of Occludin and Claudin-5 in the brain and colon tissues of MD mice decreased significantly, indicating an increase in the permeability of the intestinal and brain barriers. After treatment with the oligosaccharide esters of OE, the intestinal and brain barriers were significantly protected, and memory impairment was improved [71].

Immunohistochemical results showed that OE significantly reduced p-Tau and GFAP levels in the brains of MD mice. The increased p-Tau in the model group confirmed the successful induction of aging- and oxidative stress-related neurodegenerative changes in this memory impairment model. Hyperphosphorylation of tau protein has been reported to be associated with abnormal lipid metabolism in cognitive impairment [33]. OE administration inhibited p-Tau levels, demonstrating its ability to suppress tau phosphorylation in this model. The GFAP results indicated that OE alleviated neuroinflammation and exerted neuroprotective effects in MD mice. Collectively, these findings suggest that OE may improve memory impairment by restoring lipid metabolic homeostasis and reducing neuroinflammation in this D-galactose/AlCl₃-induced memory impairment model.

To further clarify the mechanism by which OE regulates the gut–brain axis to ameliorate memory impairment, this study employed fecal microbiota transplantation (FMT) to target the gut microbiota and investigate its mediating role in the neuroprotective effects of OE.

After establishing a mouse model of memory impairment using aluminum trichloride combined with D-galactose, the MD mice exhibited significant cognitive deficits. FMT intervention significantly shortened the escape latency in the Morris water maze, increased the time spent in the target quadrant, increased the number of platform crossings, and increased the exploration distance in the original platform area, which was consistent with the effects observed following OE intervention. These findings indicate that transplanting the gut microbiota from OE-treated mice can improve cognitive function, thereby verifying the role of the gut microbiota in OE-mediated alleviation of learning and memory impairment.

Histopathological examination of brain tissue revealed that in the model group, neurons in the hippocampal CA1, CA3, and DG regions exhibited disorganized arrangement, pronounced nuclear pyknosis, and extensive loss and dissolution of Nissl bodies, indicating neuronal damage and functional impairment. Both FMT and OE interventions significantly restored neuronal morphology and structure, increased neuronal cell count, alleviated nuclear pyknosis, and upregulated the expression level of Nissl bodies. This suggests that modulation of the gut microbiota can reduce neuronal pathology, repair brain tissue damage, and further facilitate the improvement of cognitive function.

H&E staining of colon tissue showed that model mice exhibited severe intestinal pathological damage, including epithelial shedding, inflammatory infiltration, glandular atrophy, and crypt loss. FMT significantly repaired the colonic mucosal structure, reduced inflammatory infiltration, and increased the number of goblet cells, with results consistent with those of OE intervention. This indicates that the ameliorative effect of OE on intestinal tissue damage is closely related to the regulation of the gut microbiota.

Measurement of inflammatory factors in colon and brain tissues revealed that the levels of LPS, TNF-α, and IL-6 were significantly elevated in both the colon and brain of model mice, suggesting concurrent activation of intestinal inflammation and neuroinflammation. FMT treatment reduced the levels of pro-inflammatory factors in both the colon and brain, with effects comparable to those of OE, demonstrating that the gut microbiota can achieve remote regulation of brain function by suppressing inflammation in both the colon and brain tissues. Meanwhile, FITC-dextran permeability assays confirmed that FMT significantly reduced intestinal permeability and decreased the entry of endotoxins into the bloodstream, further illustrating that intestinal barrier repair is a key link through which the gut microbiota regulates inflammation and connects the gut to the brain.

It should be noted that this study also established a group treated with a four-antibiotic cocktail to deplete the gut microbiota. The results showed that after microbiota depletion, the effects of OE in improving cognition, reducing intestinal permeability, and exerting anti-inflammatory effects were significantly weaker than those in the direct OE intervention group, demonstrating that the pharmacological effects of OE depend on the integrity of the gut microbiota. Furthermore, antibiotic treatment did not significantly affect the success rate of model establishment, indicating that the intervention protocol of this study is stable and reliable.

In conclusion, the oligosaccharide esters of Polygala tenuifolia can enhance the ability of anti-oxidative stress and neuroprotection, and improve the learning and spatial memory abilities of mice with memory impairment. Its mechanism of action may be related to improving the "gut-brain" barrier, reducing the content of brain microbiota metabolites and the levels of inflammatory factors, restoring the homeostasis of the gut microbiota, improving intestinal inflammation, and affecting glycerophospholipid metabolism.

## Limitations

This study provides evidence that OE improves memory disorder via the gut-brain axis, yet several limitations remain. First, while FMT confirmed the necessity of gut microbiota, the specific microbial taxa responsible for the therapeutic effects were not verified by monocolonization or defined consortia experiments, leaving the causality between individual bacterial species and neuroprotection unclear. Second, the D-galactose/AlCl_3_-induced model recapitulates certain aspects of cognitive impairment but does not fully represent the complex pathologies of Alzheimer's disease, such as Aβ aggregation or tau hyperphosphorylation; whether OE exerts similar effects in transgenic AD models remains unknown. Third, our multi-omics analysis revealed associations between gut microbiota, glycerophospholipid metabolites, and neuroprotective outcomes but did not establish direct molecular mechanisms. Future studies will address these gaps by performing monocolonization of key bacteria, validating OE efficacy in transgenic AD models, and conducting targeted pathway interventions to clarify causality.

## Data Availability

The data that support the findings of this study are not publicly available in a repository. However, they can be obtained from the corresponding author Huifang Li upon reasonable request, via email at lihuifangzy@sxtcm.edu.cn.

## Abbreviations

AD: Alzheimer's disease
BDNF: Brain-derived neurotrophic
CREB: Cyclic-AMP response binding protein
D-gal: D-galactose
ELISA: Enzyme-linked immunosorbent assay
GFAP: Glial Fibrillary Acidic Protein
GSH-PX: Glutathione peroxidase
H&E: Hematoxylin-eosin staining
HPLC: High-Performance Liquid Chromatography
IL-6: Interleukin-6
LEfSe: Linear discriminative analysis effect size
MDA: Malonaldehyde
MD: Memory Disorder
MWM: Morris water maze
NDD: Neurodegenerative diseases
NOR: Novel Object Recognition
OE: Oligosaccharide Ester
OTU: Operational Taxonomic Units
OS: Oxidative stress
p-Tau: Phosphorylated Tau
PTSD: Post-traumatic stress disorder
TMAO: Trimethylamine N‑oxide
TNF-α: Tumor necrosis factor α
TRKB: Tyrosine Kinase receptor B
WB: Western blot
5-HT: 5-Hydroxytryptamine

## References

[1] Liu YL, Liang XY, Zhou YY. Effects of Tenuifolin from Polygala tenuifolia on oxidative stress and inflammatory factors in PC12 cells injured by Aβ_25–35_ induction Tradit. Liaoning J Tradit Chin Med. 2022;49(7):140–3.

[2] Shi TX, Li YG, Jiang Y, Tu PF. Isolation of flavonoids from the aerial parts of *Polygala tenuifolia* Willd. and their antioxidant activities. J Chin Pharm Sci. 2013;1:36–9.

[3] Yuan HL, Gong XL, Fan Z, Liang ZG, Wang XM. Effects and mechanisms of polygalasaponins in a lipopolysaccharide-induced rat model of Parkinson’s disease. J Cap Med Univ. 2020;41:914–22.

[4] Wang XY, Liu CX, Zhou JL, et al. Research advances on chemical constituents and pharmacological effects of *Polygala tenuifolia* and predictive analysis of its potential quality markers. J Int Pharm Res. 2020;47:483–95.

[5] Niu FX, Sang XX, Yang Y, et al. Tenuifoliose O protects against β-amyloid peptide (25–35)-induced neuronal injury in SH-SY5Y cells via the AKT/CREB/BDNF signaling pathway. J Beijing Univ Tradit Chin Med. 2022;45:414–20.

[6] Wang R, Pei WH, Fang F. Polygala oligosaccharide esters ameliorate learning and memory acquisition deficits induced by scopolamine: an experimental study. J Liaoning Univ Tradit Chin Med. 2016;18:13–6.

[7] Hu Y, Liao HB, Dai-Hong G, Liu P, Wang YY, Rahman K. Antidepressant-like effects of 3,6’-disinapoyl sucrose on hippocampal neuronal plasticity and neurotrophic signal pathway in chronically mild stressed rats. Neurochem Int. 2010;56:461–5. 20018220 10.1016/j.neuint.2009.12.004

[8] Liu P, Wu L, Peng G, Han Y, Tang R, Ge J, et al. Altered microbiomes distinguish Alzheimer’s disease from amnestic mild cognitive impairment and health in a Chinese cohort. Brain Behav Immun. 2019;80:633–43. 31063846 10.1016/j.bbi.2019.05.008

[9] Liu XY, Zhang L. Spatial distribution and influencing factors of cognitive impairment among the elderly in China. Med Soc. 2025;38:1–7. 10.13723/j.yxysh.2025.09.001.

[10] Liu C. Schisandrin A ameliorates D-galactose-induced learning and memory impairment in mice: mechanisms and effects. Beihua University; 2018.

[11] Lu S, Zhao Q, Guan Y, Sun Z, Li W, Guo S, et al. The communication mechanism of the gut-brain axis and its effect on central nervous system diseases: a systematic review. Biomed Pharmacother. 2024;178:117207. 10.1016/j.biopha.2024.117207. 39067168 10.1016/j.biopha.2024.117207

[12] Cryan JF, O’Riordan KJ, Cowan CSM, Sandhu KV, Bastiaanssen TFS, Boehme M, et al. The microbiota-gut-brain axis. Physiol Rev. 2019;99:1877–2013. 31460832 10.1152/physrev.00018.2018

[13] Liu P, Hu Y, Guo DH, Lu BR, Rahman K, Mu LH, et al. Antioxidant activity of oligosaccharide ester extracted from *Polygala tenuifolia* roots in senescence-accelerated mice. Pharm Biol. 2010;48:828–33. 20645784 10.3109/13880200903283707

[14] Sun Z, Sun L, Tu L. GABAB receptor-mediated PI3K/Akt signaling pathway alleviates oxidative stress and neuronal cell injury in a rat model of Alzheimer’s disease. J Alzheimers Dis. 2020;76:1513–26. 32651311 10.3233/JAD-191032

[15] Wang JF. Investigating the protective effects and molecular mechanisms of Duchen Decoction in ameliorating cognitive impairment in aging model rats: an integrated metabolomics and microbiomics approach. Changchun University of Chinese Medicine; 2022.

[16] Sang XX. Polygala oligosaccharide esters improve learning and memory capacity and exert neuroprotective effects in an experimental mouse model of dementia. Beijing University of Chinese Medicine; 2018.

[17] Xu JQ. Improvement effect and mechanism of lotus seedpod proanthocyanidins on memory function in aged cognitively impaired rats. Huazhong University of Science and Technology; 2010.

[18] Liu Q, Xi Y, Wang Q, Liu J, Li P, Meng X, et al. Mannan oligosaccharide attenuates cognitive and behavioral disorders in the 5xFAD Alzheimer’s disease mouse model via regulating the gut microbiota-brain axis. Brain Behav Immun. 2021;95:330–43. 33839232 10.1016/j.bbi.2021.04.005

[19] Blainski A, Piccolo VK, Mello JC, de Oliveira RM. Dual effects of crude extracts obtained from *Petiveria alliacea* L. (Phytolaccaceae) on experimental anxiety in mice. J Ethnopharmacol. 2010;128:541–4. 20079419 10.1016/j.jep.2010.01.012

[20] Gao J, Bai HJ, Li Q, Li J, Wan F, Tian M, et al. In vitro investigation of the mechanism underlying the effect of ginsenoside on the proliferation and differentiation of neural stem cells subjected to oxygen-glucose deprivation/reperfusion. Int J Mol Med. 2018;41:353–63. 29138802 10.3892/ijmm.2017.3253 PMC5746305

[21] Martini F, Rosa SG, Klann IP, Fulco BCW, Carvalho FB, Rahmeier FL, et al. A multifunctional compound ebselen reverses memory impairment, apoptosis and oxidative stress in a mouse model of sporadic Alzheimer’s disease. J Psychiatr Res. 2019;109:107–17. 30521994 10.1016/j.jpsychires.2018.11.021

[22] Hu Y, Liu MY, Liu P, Dong X, Boran AD. Neuroprotective effects of 3,6’-disinapoyl sucrose through increased BDNF levels and CREB phosphorylation via the CaMKII and ERK1/2 pathway. J Mol Neurosci. 2014;53:600–7. 24488601 10.1007/s12031-013-0226-y

[23] Li C, Wang N, Zheng G, Yang L. Oral administration of resveratrol-selenium-peptide nanocomposites alleviates Alzheimer’s disease-like pathogenesis by inhibiting Aβ aggregation and regulating gut microbiota. ACS Appl Mater Interfaces. 2021;13:46406–20. 34569225 10.1021/acsami.1c14818

[24] Lissner LJ, Wartchow KM, Toniazzo AP, Gonçalves CA, Rodrigues L. Object recognition and Morris water maze to detect cognitive impairment from mild hippocampal damage in rats: a reflection based on the literature and experience. Pharmacol Biochem Behav. 2021;210:173273. 34536480 10.1016/j.pbb.2021.173273

[25] Rosa E, Fahnestock M. CREB expression mediates amyloid β-induced basal BDNF downregulation. Neurobiol Aging. 2015;36:2406–13. 26025137 10.1016/j.neurobiolaging.2015.04.014

[26] Harward SC, Hedrick NG, Hall CE, Parra-Bueno P, Milner TA, Pan E, et al. Autocrine BDNF-TrkB signalling within a single dendritic spine. Nature. 2016;538:99–103. 27680698 10.1038/nature19766 PMC5398094

[27] Bonni A, Brunet A, West AE, Datta SR, Takasu MA, Greenberg ME. Cell survival promoted by the Ras-MAPK signaling pathway by transcription-dependent and -independent mechanisms. Science. 1999;286:1358–62. 10558990 10.1126/science.286.5443.1358

[28] Mehrafza S, Kermanshahi S, Mostafidi S, et al. Pharmacological evidence for lithium-induced neuroprotection against methamphetamine-induced neurodegeneration via Akt-1/GSK3 and CREB-BDNF signaling pathways. Iran J Basic Med Sci. 2019;22:856–65. 31579440 10.22038/ijbms.2019.30855.7442 PMC6760490

[29] Jin J, Xu Z, Zhang L, Zhang C, Zhao X, Mao Y, et al. Gut-derived β-amyloid: likely a centerpiece of the gut-brain axis contributing to Alzheimer’s pathogenesis. Gut Microbes. 2023;15:2167172. 36683147 10.1080/19490976.2023.2167172 PMC9872956

[30] Vogt NM, Kerby RL, Dill-McFarland KA, Harding SJ, Merluzzi AP, Johnson SC, et al. Gut microbiome alterations in Alzheimer’s disease. Sci Rep. 2017;7:13537. 29051531 10.1038/s41598-017-13601-y PMC5648830

[31] Meyer K, Lulla A, Debroy K, Shikany JM, Yaffe K, Meirelles O, et al. Association of the gut microbiota with cognitive function in midlife. JAMA Netw Open. 2022;5:e2143941. 35133436 10.1001/jamanetworkopen.2021.43941 PMC8826173

[32] Yin F. Lipid metabolism and Alzheimer’s disease: clinical evidence, mechanistic link and therapeutic promise. FEBS J. 2023;290:1420–53. 34997690 10.1111/febs.16344 PMC9259766

[33] Tong B, Ba Y, Li Z, Yang C, Su K, Qi H, et al. Targeting dysregulated lipid metabolism for the treatment of Alzheimer’s disease and Parkinson’s disease: current advancements and future prospects. Neurobiol Dis. 2024;196:106505. 38642715 10.1016/j.nbd.2024.106505

[34] Hamilton JA, Hillard CJ, Spector AA, Watkins PA. Brain uptake and utilization of fatty acids, lipids and lipoproteins: application to neurological disorders. J Mol Neurosci. 2007;33:2–11. 17901539 10.1007/s12031-007-0060-1

[35] Chung JY, Kim W, Im W, Yoo DY, Choi JH, Hwang IK, et al. Neuroprotective effects of adipose-derived stem cells against ischemic neuronal damage in the rabbit spinal cord. J Neurol Sci. 2012;317:40–6. 22475376 10.1016/j.jns.2012.02.035

[36] Mesa-Herrera F, Taoro-González L, Valdés-Baizabal C, Diaz M, Marín R. Lipid and lipid raft alteration in aging and neurodegenerative diseases: a window for the development of new biomarkers. Int J Mol Sci. 2019;20:3810. 31382686 10.3390/ijms20153810 PMC6696273

[37] Ng YW, Say YH. Palmitic acid induces neurotoxicity and gliatoxicity in SH-SY5Y human neuroblastoma and T98G human glioblastoma cells. PeerJ. 2018;6:e4696. 29713567 10.7717/peerj.4696 PMC5924683

[38] Fabelo N, Martín V, Santpere G, Marín R, Torrent L, Ferrer I, et al. Severe alterations in lipid composition of frontal cortex lipid rafts from Parkinson’s disease and incidental Parkinson’s disease. Mol Med. 2011;17:1107–18. 21717034 10.2119/molmed.2011.00119 PMC3188884

[39] Melo HM, Seixas da Silva GDS, Sant’Ana MR, Teixeira CVL, Clarke JR, Miya Coreixas VS, et al. Palmitate is increased in the cerebrospinal fluid of humans with obesity and induces memory impairment in mice via pro-inflammatory TNF-α. Cell Rep. 2020;30:2180-2194.e8. 32075735 10.1016/j.celrep.2020.01.072

[40] Ma HG, Pan QM, Zhu FF, Wang YX. Correlation analysis between leukotriene B4 and progressive ischemic stroke caused by cerebral arteriosclerosis. Clin Educ Gen Pract. 2017;15:153–5.

[41] Fang MF, Tan F, Wang XW. Inhibition of cytokine signaling 3 and the correlation between leukotriene B4 and interleukin 6 with infarct volume. Zhonghua Lao Nian Xin Nao Xue Guan Bing Za Zhi. 2018;20:1095–6.

[42] Sheikh AM, Michikawa M, Kim SU, Nagai A. Lysophosphatidylcholine increases the neurotoxicity of Alzheimer’s amyloid β1-42 peptide: role of oligomer formation. Neuroscience. 2015;292:159–69. 25727637 10.1016/j.neuroscience.2015.02.034

[43] Tzekov R, Dawson C, Orlando M, Mouzon B, Reed J, Evans J, et al. Sub-chronic neuropathological and biochemical changes in mouse visual system after repetitive mild traumatic brain injury. PLoS ONE. 2016;11:e0153608. 27088355 10.1371/journal.pone.0153608 PMC4835061

[44] Lin W, Zhang J, Liu Y, Wu R, Yang H, Hu X, et al. Studies on diagnostic biomarkers and therapeutic mechanism of Alzheimer’s disease through metabolomics and hippocampal proteomics. Eur J Pharm Sci. 2017;105:119–26. 28495476 10.1016/j.ejps.2017.05.003

[45] Mapstone M, Cheema AK, Fiandaca MS, Zhong X, Mhyre TR, MacArthur LH, et al. Plasma phospholipids identify antecedent memory impairment in older adults. Nat Med. 2014;20:415–8. 24608097 10.1038/nm.3466 PMC5360460

[46] Narukawa M, Kamiyoshihara A, Izu H, Fujii T, Matsubara K, Misaka T. Efficacy of long-term feeding of α-glycerophosphocholine for aging-related phenomena in old mice. Gerontology. 2020;66:275–85. 31968334 10.1159/000504962

[47] Mohammadi M, Manaheji H, Maghsoudi N, Danyali S, Baniasadi M, Zaringhalam J. Microglia dependent BDNF and proBDNF can impair spatial memory performance during persistent inflammatory pain. Behav Brain Res. 2020;390:112683. 32442548 10.1016/j.bbr.2020.112683

[48] Song AQ, Gao B, Fan JJ, Zhu YJ, Zhou J, Wang YL, et al. NLRP1 inflammasome contributes to chronic stress-induced depressive-like behaviors in mice. J Neuroinflammation. 2020;17:178. 32513185 10.1186/s12974-020-01848-8 PMC7281929

[49] Dmytriv TR, Storey KB, Lushchak VI. Intestinal barrier permeability: the influence of gut microbiota, nutrition, and exercise. Front Physiol. 2024;15:1380713. 39040079 10.3389/fphys.2024.1380713 PMC11260943

[50] Ma J, Piao X, Mahfuz S, Long S, Wang J. The interaction among gut microbes, the intestinal barrier and short chain fatty acids. Anim Nutr. 2021;9:159–74. 35573092 10.1016/j.aninu.2021.09.012 PMC9079705

[51] Molloy MJ, Bouladoux N, Belkaid Y. Intestinal microbiota: shaping local and systemic immune responses. Semin Immunol. 2012;24:58–66. 22178452 10.1016/j.smim.2011.11.008 PMC3292882

[52] Wang L, Zhu L, Qin S. Gut microbiota modulation on intestinal mucosal adaptive immunity. J Immunol Res. 2019;2019:4735040. 31687412 10.1155/2019/4735040 PMC6794961

[53] Liu T, Zhang L, Joo D, Sun SC. NF-κB signaling in inflammation. Signal Transduct Target Ther. 2017;2:17023. 29158945 10.1038/sigtrans.2017.23 PMC5661633

[54] Di Vincenzo F, Del Gaudio A, Petito V, Lopetuso LR, Scaldaferri F. Gut microbiota, intestinal permeability, and systemic inflammation: a narrative review. Intern Emerg Med. 2024;19:275–93. 37505311 10.1007/s11739-023-03374-w PMC10954893

[55] Huang X, Hussain B, Chang J. Peripheral inflammation and blood-brain barrier disruption: effects and mechanisms. CNS Neurosci Ther. 2021;27:36–47. 33381913 10.1111/cns.13569 PMC7804893

[56] Shao F, Wang X, Wu H, Wu Q, Zhang J. Microglia and neuroinflammation: crucial pathological mechanisms in traumatic brain injury-induced neurodegeneration. Front Aging Neurosci. 2022;14:825086. 35401152 10.3389/fnagi.2022.825086 PMC8990307

[57] Kölliker-Frers R, Udovin L, Otero-Losada M, Kobiec T, Herrera MI, Palacios J, et al. Neuroinflammation: an integrating overview of reactive-neuroimmune cell interactions in health and disease. Mediators Inflamm. 2021;2021:9999146. 34158806 10.1155/2021/9999146 PMC8187052

[58] Müller L, Di Benedetto S, Müller V. From homeostasis to neuroinflammation: insights into cellular and molecular interactions and network dynamics. Cells. 2025;14:54. 39791755 10.3390/cells14010054 PMC11720143

[59] Mbiydzenyuy NE, Qulu LA. Stress, hypothalamic-pituitary-adrenal axis, hypothalamic-pituitary-gonadal axis, and aggression. Metab Brain Dis. 2024;39:1613–36. 39083184 10.1007/s11011-024-01393-w PMC11535056

[60] Sugama S, Kakinuma Y. Stress and brain immunity: microglial homeostasis through hypothalamus-pituitary-adrenal gland axis and sympathetic nervous system. Brain Behav Immun. 2020;7:100111. 10.1016/j.bbih.2020.100111 PMC8474505 34589871

[61] Qu S, Yu Z, Zhou Y, Wang S, Jia M, Chen T, et al. Gut microbiota modulates neurotransmitter and gut-brain signaling. Microbiol Res. 2024;287:127858. 39106786 10.1016/j.micres.2024.127858

[62] Sacks D, Baxter B, Campbell BCV, Carpenter JS, Cognard C, Dippel D, et al. Multisociety consensus quality improvement revised consensus statement for endovascular therapy of acute ischemic stroke. Int J Stroke. 2018;13:612–32. 29786478 10.1177/1747493018778713

[63] Nagpal R, Neth BJ, Wang S, Craft S, Yadav H. Modified Mediterranean-ketogenic diet modulates gut microbiome and short-chain fatty acids in association with Alzheimer’s disease markers in subjects with mild cognitive impairment. EBioMedicine. 2019;47:529–42. 31477562 10.1016/j.ebiom.2019.08.032 PMC6796564

[64] D’Argenio V, Sarnataro D. Microbiome influence in the pathogenesis of prion and Alzheimer’s diseases. Int J Mol Sci. 2019;20:4704. 31547531 10.3390/ijms20194704 PMC6801937

[65] Ciranna L. Serotonin as a modulator of glutamate- and GABA-mediated neurotransmission: implications in physiological functions and in pathology. Curr Neuropharmacol. 2006;4:101–14. 18615128 10.2174/157015906776359540 PMC2430669

[66] Sun MF, Ouyang JF, Wu CY, Cheng JJ. Mechanism of wogonin in alleviating LPS-induced inflammation in BV-2 cells and protecting SH-SY5Y cells. Chin J Exp Tradit Med Form. 2024;30:62–9.

[67] Zhao Y, Jaber V, Lukiw WJ. Secretory products of the human GI tract microbiome and their potential impact on Alzheimer’s disease (AD): detection of lipopolysaccharide (LPS) in AD hippocampus. Front Cell Infect Microbiol. 2017;7:318. 28744452 10.3389/fcimb.2017.00318 PMC5504724

[68] Benakis C, Martin-Gallausiaux C, Trezzi JP, Melton P, Liesz A, Wilmes P. The microbiome-gut-brain axis in acute and chronic brain diseases. Curr Opin Neurobiol. 2020;61:1–9. 31812830 10.1016/j.conb.2019.11.009

[69] Zhu A, Wang Q. Cellular and molecular mechanisms of blood-brain barrier permeability alteration: a review of recent advances. Chin J Pharmacol Toxicol. 2017;31:889–99.

[70] Phillips JM, Winfree RL, Seto M, Schneider JA, Bennett DA, Dumitrescu LC, et al. Pathologic and clinical correlates of region-specific brain GFAP in Alzheimer’s disease. Acta Neuropathol. 2024;148:69. 39580758 10.1007/s00401-024-02828-5 PMC11586308

[71] Bourassa MW, Alim I, Bultman SJ, Ratan RR. Butyrate, neuroepigenetics and the gut microbiome: can a high fiber diet improve brain health? Neurosci Lett. 2016;625:56–63. 26868600 10.1016/j.neulet.2016.02.009 PMC4903954

